# Proteomic study revealed antipsychotics-induced nuclear protein regulations in B35 cells are similar to the regulations in C6 cells and rat cortex

**DOI:** 10.1186/s40360-018-0199-0

**Published:** 2018-03-07

**Authors:** Tinchou Li, Mingcheng Lee, Fuming Tsai, Yunhsiang Chen, Yiyin Lin, Maoliang Chen

**Affiliations:** 1 Division of Neurosurgery, Department of Surgery, Taipei Tzu Chi Hospital, Buddhist Tzu Chi Medical Foundation, New Taipei City, Taiwan, Republic of China; 2 Department of Surgery, School of Medicine, Tzu Chi University, Hualien City, Taiwan, Republic of China; 3 Department of Research, Taipei Tzu Chi Hospital, Buddhist Tzu Chi Medical Foundation, New Taipei City, Taiwan, Republic of China; 4 Department of Microbiology, Soochow University, Shih Lin, Taipei City, Taiwan, Republic of China; 5 Department of Life Science, Fu Jen Catholic University, New Taipei City, Taiwan, Republic of China

**Keywords:** Antipsychotic drugs (APDs) - nuclear protein - rat prefrontal cortex - neuronal cell - glial cell

## Abstract

**Background:**

Based on accumulating evidence, the regulation of protein expression by antipsychotic drugs (APDs) might be closely related to the control of psychotic symptoms when these drugs are used to treat mental disorders. The low quantity of nuclear proteins in the cell hinders their detection because signal for rare proteins are masked in most proteomic detection systems.

**Methods:**

Nuclear proteins fractionated from APD-treated B35 cells were labeled with iTRAQ and detected by LC/MS/MS to investigate APD-induced alterations in nuclear protein expression. Western blot, immunofluorescent cell staining, and immunohistochemical staining were applied to validate the findings.

**Results:**

The expression of ADP/ATP translocase 2, heat shock cognate 71 kDa protein, histone H1.2, histone H3.3, histone H4, non-POU domain-containing octamer-binding protein, nucleolin, nucleophosmin, prelamin-A/C, plectin-1, vimentin, and 40S ribosomal protein S3a was regulated by APDs in B35 cells, according to our proteomic data. According to the results of the gene ontology analysis, all these proteins played important roles in biological processes or in molecular functions in cells. Western blot results showing APD-induced alterations in nuclear protein expression in B35 cells were consistent with the LC/MS/MS results. Heat shock cognate 71 kDa protein and vimentin expression in C6 cells were not affected by the three APDs. As shown in the immunofluorescent cell staining, all the three APDs altered protein expression to similar extents. We also examined whether the expression of these proteins was affected by APDs in the prefrontal cortex of rats administered sub-chronic and chronic APD treatments by western blotting and immunohistochemical staining.

**Conclusions:**

The findings of the proteomic analysis of APD-treated B35 cells were recapitulated in the APD-treated rat cortex. The expression of some proteins was altered by APDs in rat prefrontal cortex in a time-dependent manner.

**Electronic supplementary material:**

The online version of this article (10.1186/s40360-018-0199-0) contains supplementary material, which is available to authorized users.

## Background

Antipsychotic drugs (APDs) have been proposed to regulate gene and protein expression in the brain [1–3]. Based on accumulating evidence, APDs also ease psychotic symptoms and induce side-effects by regulating gene or protein expression levels [3]. Most psychotic disorders, including schizophrenia, are genetically complex diseases with unclear pathogenic mechanisms. In addition, the complexity of actions of antipsychotic drugs on regulating psychotic symptoms and other drug effects originating from the variety of binding profiles of each APD [4] has hampered our ability to clarify the molecular mechanisms underlying the actions of antipsychotic drugs.

Recently, studies of DNA-methylation, post-translational modifications (PTMs) of histone proteins and non-coding RNA regulated protein translation have suggested that proteins in cell nuclei might play important roles in the epigenetic regulation of cell functions [5, 6]. In addition, proteins’ functions related to the regulations of gene and protein expression in cell nuclei are also major targets for studying the mechanism of the actions of APDs and aetiology of mental disorders.

O’Brien and colleagues used 2-dimensional polyacrylamide gel electrophoresis (2D-PAGE) to discover the differential protein expression profiles between the risperidone-treated and control rat striatum and to reveal the possible causes of extrapyramidal symptoms (EPS) induced by antipsychotics [7]. Ji and colleagues also used 2D-PAGE to examine the effects of chlorpromazine, clozapine, quetiapine on rat mitochondria from the rat cerebral cortex and hippocampus and found that abnormal cerebral energy metabolism might be involved in the both the curative effects and side effects of antipsychotics [8]. Kedracka-Krok and colleagues used 2-dimensional difference gel electrophoresis (2D-DIGE) to examine protein expression profiles in the cerebral cortex of rats treated with clozapine or risperidone [9]. Clozapine was shown to regulate proteins related to the cytoskeletal structure and calcium homeostasis.

Liquid chromatography-tandem mass spectrometry (LC/MS/MS) has been widely applied in proteomic studies over the last two decades to identify possible biomarkers for studying various diseases [10–12]. LC/MS/MS separates and detects digested peptides to identify detected proteins utilizing peptide spectrum algorithms in a peptide identification database. It also precedes relative quantification with or without isotope labelling to determine and compare the expression of proteins in different samples [10]. However, the complexity of the sample used for the analysis represents a critical issue in the ability to produce reliable detection results, even when a sensitive and accurate LC/MS/MS platform that has been improved by developing detection techniques is available. Another breakthrough is the use of stable isotope reagents, such as isobaric tags for relative and absolute quantitation (iTRAQ) reagents [13, 14], which were developed for labelling peptides to enable multiplex sample analysis during LC/MS/MS detection processes [15, 16]. These stable isotope reagents enable the identification and quantification of multiple samples in one detection run to reduce the possible bias between batches.

The enrichment of particular types of proteins, such as glycoproteins (glycome) or phosphoproteins (phosphoproteome) [17, 18], is another way to discover the specific low abundance proteins in a crude protein mixture in study of schizophrenia. The fractionation of proteins according to their molecular properties or their location in cells also reduces the sample complexity. In this study, nuclear proteins purified from APD-treated B35 were labelled with iTRAQ reagents and analysed by LC/MS/MS to discover the regulatory effects of APDs on protein expression in nuclei. Differentially expressed nuclear proteins detected by LC/MS/MS were then further validated in APD-treated B35 cells, C6 cells and the rat prefrontal cortex by western blotting, immunofluorescent staining and/or immunohistochemical staining.

## Methods

### Antibodies

The antibodies included: anti-actin, ab6276 (Abcam, Cambridge, UK); anti-Histone H4 (HIST1H4B), #13919 (Cell Signaling Technology, Danvers, MA, USA); anti- Heat shock cognate 71 kDa protein (HSPA8), #8444 (Cell Signaling Technology); anti-nucleolin (NCL), #12247 (Cell Signaling Technology); anti-nucleophosmin (NPM1), #3542 (Cell Signaling Technology); anti-vimentin (VIM), #3932 (Cell Signaling Technology); anti-plectin-1 (PLEC), #12254 (Cell Signaling Technology). All antibodies were used at the recommended fold dilution described on the manufacture’s datasheet.

### Cell culture and APD treatments

Risperidone was obtained from Janssen-Pharmaceutica (Beerse, Belgium), and haloperidol and clozapine were obtained from Sigma-Aldrich (St. Louis, MO, USA). C6 and B35 cells were obtained from the Bioresource Collection and Research Center (BCRC) of the Food Industry Research and Development Institute (FRDI), Taiwan. C6 cells were cultured in DMEM high-glucose medium (Invitrogen Life Technologies, Carlsbad, CA, USA) supplemented with 2 mM L-glutamine, 10% horse serum (Invitrogen Life Technologies), and 2% fetal bovine serum (Invitrogen Life Technologies). B35 cells were maintained in MEM medium (Invitrogen Life Technologies) supplemented with 10% fetal bovine serum (Invitrogen Life Technologies). One of the APDs (haloperidol, risperidone, or clozapine) was added to the medium once a day. The concentrations of each APD used in this study were determined based on results from our previous publication. A 1 × PBS solution was added to culture medium to serve as the control supplement. The final concentration of each APD in the culture medium was 4 μg/ml (10 μM) for haloperidol, 4 μg/ml (8 μM) for risperidone, and 2.5 μg/ml (7.65 μM) for clozapine. C6 cells were subcultured when they exhibited a density of approximately 70% confluence to prevent abnormal production of the S100 protein. Cells were harvested when they had reached 60–70% confluence after treatment with APDs for 5 days.

### Animals and drug treatment

All experimental procedures used to treat animals in this study were approved by the Institutional Animal Care and Use Committee (IACUC) of Taipei Tzu Chi Hospital, Buddhist Tzu Chi Medical Foundation (101-IACUC-028). Male Sprague-Dawley (SD) rats weighing 120–150 g were housed in a temperature- and humidity-controlled feeding room with a 12-h light/ dark cycle and had free access to water and food for 5 days before the APD treatment. Two independent APD treatment batches were performed in this study. In both batches, rats (*n* = 3 per group) were intraperitoneally injected with haloperidol (1 mg/kg), risperidone (1 mg/kg), clozapine (20 mg/kg), or PBS once daily for 7 days for sub-chronic APD treatment. For the chronic treatment, two independent APD treatment batches (n = 3 per group in each batch) of SD rats received haloperidol (1 mg/kg), risperidone (1 mg/kg), clozapine (20 mg/kg), or vehicle once daily for 28 days. SD rats were sacrificed by CO_2_ asphyxiation 24 h after the last injection. Dissected cerebral cortices were stored in liquid nitrogen or were soaked in 4% paraformaldehyde for 24 h for paraffin embedding.

### Nuclear protein extraction

C6 or B35 cells treated with each APD were harvested for nuclear protein extraction using a Nuclear Extraction Kit (Chemicon International Inc., California, USA), according to manufacture’s instructions. Briefly, cells were harvested and were resuspended in 1 × Cytoplasmic Lysis Buffer containing 0.5 mM DTT and a Protease Inhibitor Mix (GE Healthcare Bio-Science, Uppsala, Sweden). The mixture was then placed on ice for 15 min and centrifuged at 250×g for 5 min at 4 °C. The cell pellet was resuspended in 1 × Cytoplasmic Lysis Buffer and the cells were disrupted using a syringe with a 27-gauge needle followed by centrifugation at 8000×g for 20 min at 4 °C. The supernatant was collected as the cytoplasmic proteins fraction. The nuclear pellet was washed and lysed with Nuclear Extraction Buffer at 4 °C for 60 min, and the nuclear extract was then centrifuged at 16,000×g for 5 min at 4 °C. The supernatant was transferred to a fresh tube and served as the nuclear protein fraction.

### iTRAQ labelling and LC/MS/MS proteomic analysis

Fractionated nuclear proteins from APD-treated B35 cells were labelled with iTRAQ reagents (Applied Biosystems), according to the manufacturer’s instructions. Briefly, 10 μg of nuclear proteins from B35 cells were reduced with a reducing reagent at 60 °C for 1 h. Cysteine residues were then blocked with cysteine blocking reagent for 10 min at room temperature, followed by trypsin digestion at 37 °C for 16 h. iTRAQ-4 plex labelling reagents were then added to each peptide sample and the mixtures were further incubated at room temperature for 60 min. Each of the peptide samples was then combined and evaporated in a vacuum dryer at room temperature. The dried residue was dissolved in 50 μl of ddH_2_O. Five microliters of iTRAQ-labelled residue were analysed using an HPLC system (Agilent) coupled with a 3200 Q TRAP LC/MS/MS System (MDS SCIEX, Applied Biosystems). The iTRAQ-labelled residue was separated on the reverse-phase C18 column at a flow rate of 10 μl/min. The eluent gradient consisted of buffer A (0.1% formic acid in H_2_O) and buffer B (0.1% formic acid in acetonitrile). Peptides were subjected to electrospray ionization followed by tandem mass spectrometry. The collected data were then analysed to identify and quantify the proteins using the ProteinPilot ™ 3.0 (MDS SCIEX) software.

### Western blot analysis

Nuclear protein extracts from both cell lines and the rat cerebral cortex were prepared as described above. Approximately 5–20 μg of nuclear proteins were separated on 12.5% sodium dodecyl sulphate polyacrylamide gels. The resolved proteins in the gel were then transferred to polyvinylidene difluoride membranes. After blocking, membranes were incubated with specific primary antibodies for 14 h at 4 °C. After washes with 1 × PBS, membranes were then incubated with horseradish peroxidase-conjugated goat anti-mouse or anti-rabbit antibodies (Cat. # 401215 and # 401315, Calbiochem, Darmstadt, Germany) at room temperature for 1 h. Target protein bands were developed using the Amersham ECL kit (Amersham, Bucks, UK). We examined the lack of GAPDH in the nuclear fraction and the lack of lamin B in the cytosolic fraction to control the quality of nuclear extraction processes. Beta-actin was examined served as a quantification control.

### Immunofluorescent staining of C6 and B35 cells

C6 or B35 cells were seeded onto coverslips in a 6-well plate at a density of 5 × 10^3^ cells per well and cultured in medium for 5 days with APDs added daily as described in the drug treatment section above. After APD treatment, coverslips with C6 or B35 cells were transferred to a fresh 6-well plate and washed with 1× PBS. Cells were then fixed by incubating the coverslips in paraformaldehyde for 15 min. Coverslips were then washed with 1× PBS. Cells were further permeabilized with methanol and incubated at − 20 °C for 15 min. After blocking with BSA for 15 min at room temperature, coverslips were incubated with a specific antibody for 1 h at room temperature. Coverslips were washed three times with 0.1% PBST and then incubated with specific secondary antibodies for 1 h at room temperature. Antibodies were removed and the coverslips were washed with 0.1% PBST followed by 4′6-diamidino-2-phenylindole (DAPI) staining for 4 min. Coverslips were then washed with 1 × PBS, mounted on slides with mounting gel, and sealed with nail polish. Quantification of the fluorescent intensity was performed using the image quantification software ImageJ 1.50i obtained from the NIH website (http://imagej.nih.gov/ij/). Areas of interest on the image were selected using the polygon selection method to quantify the immunofluorescent staining. The mean intensities of all areas were then calculated to represent the expression level of the detected protein.

### Immunohistochemistry analysis of rat prefrontal cortex

Paraffin-embedded brain samples were sectioned at 5 μm and placed on silane-coated glass slides. Slides containing sections were incubated at 70 °C for 2 h and then immediately immersed in Trilogy solution (Cell Marque, BH, The Hague, NL) to remove the paraffin. Slides were pressure heated in Trilogy solution at 120 °C for 15 min to remove the traces of paraffin and to retrieval the antigen in the samples. After washing the slides twice with ddH_2_O, tissue sections were incubated with 3% H_2_O_2_ in PBS to eliminate endogenous peroxidase activity. Sections were then blocked with 2% BSA in PBS for 20 min and then incubated with a specific antibody for 2 h at 37 °C. Slides were washed twice with PBS and incubated with a specific secondary antibody for 30 min at room temperature. Slides were washed twice with PBS, followed by application of the substrate solution, 3,3′-diaminobenzidine tetrahydrochloride (DAB) to develop the result. The developing reaction was stopped by immersing the slides in ddH_2_O twice, followed by the addition of Mayer’s haematoxylin for counterstaining (Thermo Fisher Scientific). Slides were then rinsed with ddH_2_O three times, cleared in a non-xylene solution, and mounted with coverslips and the slide mounting medium DPX Mountant (Sigma). For determining changes in protein expression in the immunohistochemistry analysis, two independent colleagues were invited to compare the changes in protein expression on images of the immunohistochemical staining.

### Total RNA isolation and gene expression assay

Total RNA was extracted from sub-chronic APD-treated C6 or B35 cells using TRIZOL Total RNA Isolation Reagent (Invitrogen Life Technologies), according to the manufacturer’s instructions. Briefly, cells were directly lysed and homogenized in 1 ml of TRIZOL denaturing reagent in a 10-cm cell culture dish followed by transfer to 1.5-ml centrifuge tubes. Homogenates were incubated on ice for 5 min, vortexed vigorously, and centrifuged at 12,000 x g for 10 min at 4 °C. Supernatants were then transferred to fresh tubes. Two hundred microliters of chloroform were added to each ml of supernatant, and the samples were then vigorously mixed and incubated on ice for 5 min, followed by centrifugation at 12,000 x g for 10 min at 4 °C. Each aqueous phase was transferred to a fresh tube, and the chloroform extraction procedure was repeated. Each aqueous phase was collected and mixed with 0.7 volume of 2-propanol. The mixtures were incubated at − 20 °C for 1 h and centrifuged at 12,000 x g for 30 min at 4 °C. RNA pellets were washed twice with 75% ethanol, air dried, and dissolved in RNase-free water. Reverse transcription was performed using High Capacity cDNA Reverse Transcription Kits (Applied Biosystems), according to the manufacturer’s instructions. Real-time quantitative polymerase chain reactions (RT-QPCR) were performed using an ABI 7900 HT Fast Real-Time PCR System in combination with continuous SYBR Green detection (Applied Biosystems) after the reverse transcription reaction. RT-QPCR was performed in a reaction volume of 20 μl containing 2 μl of diluted cDNAs, 10 μl of SYBR Green PCR Master Mix (Applied Biosystems), 2 μl each of sense and antisense primers (2 mM), and 4 μl of H_2_O. The primer sequences for each gene used in this study are listed in Additional file 1: Table S1. The general PCR conditions were: polymerase activation at 95 °C for 10 min followed by 50 cycles of denaturation at 95 °C for 15 s, annealing at 60 °C for 30 s, and extension at 72 °C for 60 s. After amplification, a melting curve was acquired to determine the optimal PCR conditions. The relative standard curve method was used to quantify mRNA expression. The relative gene expression levels in each sample were calculated from the respective standard curves using a linear regression analysis. The mRNA expression levels of each gene were normalized to beta-actin expression. All experiments were performed in duplicate. Differences in the normalized mRNA expression levels of the examined gene between APD-treated and drug-naive C6 or B35 cells were assessed using one-way ANOVA followed by the Dunnett post hoc comparison test. One-way ANOVA and the Dunnett post hoc comparison test were performed with SPSS Statistics 17.0.

## Results

### LC/MS/MS revealed the regulation of protein expression in the nuclei of APD-treated B35 cells

Nuclear proteins harvested from each of the APDs-treated B35 and control B35 cells were labelled with iTRAQ reagents and were analysed using LC/MS/MS. Three independent batches of nuclear proteins from APD-treated B35 cells were used as biological repeats. Twenty-five proteins were identified by LC/MS/MS. As shown in Table 1, ADP/ATP translocase 2(SLC25A5), heat shock cognate 71 kDa protein (HSPA8), histone H1.2 (HIST1H1C), histone H3.3(H3F3B), histone H4(HIST1H4B), non-POU domain-containing octamer-binding protein(NONO), nucleolin(NCL), nucleophosmin(NPM1), prelamin-A/C(LMNA), plectin-1(PLEC), vimentin(VIM), and 40S ribosomal protein S3a(RPS3A) represent 12 proteins that showed significant changes (*p*-value< 0.05) in expression in B35 cells treated with at least one of the three APDs. According to the gene ontology analysis, all these proteins played important roles in biological processes in cells (Table 2). Some proteins play roles in molecular functions or as cellular components. HIST1H1C, H3F3B, and HIST1H4B are histone proteins. HSPA8 and NPM1 are chaperone proteins in cells. SLC25A5 and RPS3A are ribosomal proteins that regulate ribosome function. LMNA and VIM are structural proteins and act as intermediate filaments in cells.

**Table 1:** List of proteins identified by LC/MS/MS that were differentially regulated by various APDs in B35 cells

UniProt ID	Name	Fold change compared to control group		
		HAL	RIS	CLO
Q09073	ADP/ATP translocase 2	1.604	1.765	0.967
P63018	Heat shock cognate 71 kDa protein	2.644	3.754	1.414
P15865	Histone H1.2	1.791	1.971	1.182
P84245	Histone H3.3	1.510	1.319	0.539
P62804	Histone H4	1.553	1.235	0.528
Q5FVM4	Non-POU domain-containing octamer-binding protein	1.582	1.428	1.009
P13383	Nucleolin	2.147	2.294	1.527
P13084	Nucleophosmin	2.233	2.651	1.650
P30427	Plectin-1	2.156	2.158	1.230
P48679	Prelamin-A/C	1.775	1.865	1.302
P31000	Vimentin	2.130	2.369	1.593
P49242	40S ribosomal protein S3a	2.238	2.479	1.833

**Table 2:** Gene ontology analysis of nuclear proteins identified in APD-treated B35 cells

Name	Protein Class	Function Class
Histone H4 (HIST1H4B)	histone	Biological Process
Histone H3.3 (H3F3B)	histone	Biological Process
ADP/ATP translocase 2 (SLC25A5)	amino acid transportermitochondrial carrier proteintransfer/carrier proteinribosomal proteincalmodulin	Molecular Function Biological Process Cellular Component
Non-POU domain-containing octamer-binding protein (NONO)	mRNA splicing factor	Molecular Function Biological Process
Histone H1.2 (HIST1H1C)	histone	Biological Process
Plectin-1 (PLEC)	non-motor actin binding protein	Molecular Function Biological Process Cellular Component
Prelamin-A/C (LMNA)	structural protein intermediate filament	Molecular Function Biological Process
Heat shock cognate 71 kDa protein (HSPA8)	Hsp70 family protein chaperone	Biological Process
Nucleolin (NCL)	–	Biological Process
Vimentin (VIM)	structural protein intermediate filament	Molecular Function Biological Process Cellular Component
Nucleophosmin (NPM1)	chaperone	Biological Process
40S ribosomal protein S3a (RPS3A)	ribosomal protein	Molecular Function Biological Process Cellular Component

### Validation of APD-induced regulation of protein expression in the nuclei of B35 and C6 cells

**Fig. 1:**
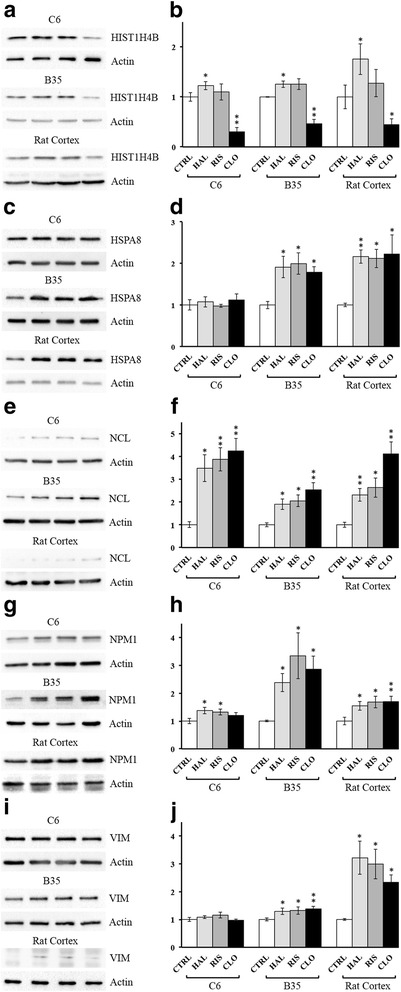
Western blot showing APD-induced changes in the expression of the HIST1H4B, HSPA8, NCL, NPM1, and VIM proteins in C6 and B35 cells and the rat cortex. Nuclear protein extracts collected from APD-treated C6 cells, B35 cells and rat cortex were compared. **a** and (**b**), HIST1H4B; (**c**) and (**d**),HSPA8; (**e**) and (**f**), NCL; (**g**) and (**h**), NPM1; (**i**) and (**j**), VIM. Bar charts were constructed from triplicate western blot data obtained from three different batches of APD-treated cells or rat cortices using ANOVA followed by Dunnett’s test (*p-value < 0.05; **p-value < 0.01). Haloperidol (HAL), risperidone (RIS), clozapine (CLO), or PBS (control group, CTRL))

Our previous researches mentioned that APDs might induce differential protein expression regulation in different cell types, such as B35 neuronal cells and C6 glial cells. We then further validated APD-induced regulation of protein expression in the nuclei of B35 and C6 cells. Five proteins were selected for validation using western blotting. Almost all selected targets showed consistent changes in expression in the nuclei of APD-treated B35 cells as measured using LC/MS/MS (Fig. 1(**a**)-(**j**)). Only HIST1H4B expression in B35 nuclei was not affected by risperidone (Fig. 1(**a**) and (**b**)). In C6 nuclei, we found that HSPA8 (Fig. 1(**c**) and (**d**)) and VIM (Fig. 1(**i**) and (**j**)) expression were not affected by any of the three APDs. HIST1H4B (Fig. 1(**a**) and (**b**)) and NCL (Fig. 1(**e**) and (**f**)) expression in C6 nuclei were regulated by APDs, similar to APD-treated B35 cells. Haloperidol and risperidone induced NPM1 expression in C6 nuclei (Fig. 1(**g**) and (**h**)). Clozapine did not significantly affect NPM1 expression in C6 nuclei.

### Western blots revealed differences in protein expression in the sub-chronic APD-treated rat cortex

We also examined HIST1H4B, HSPA8, NCL, NPM1, and VIM expression in APD-treated rat cortices. As shown in Fig. 1, HSPA8, NCL, NPM1, and VIM expression were upregulated in the rat cortex following treatment with any of the three APDs. HIST1H4B expression in the rat cortex was upregulated by haloperidol but reduced by clozapine (Fig. 1(**a**) and (**b**)). The sub-chronic risperidone treatment did not affect HIST1H4B expression in the rat cortex.

### Immunofluorescent staining revealed APD-induced nuclear protein regulation in C6 and B35 cells

Immunofluorescent staining was also used to examine the regulation of protein expression by APD treatments in C6 and B35 cells. In both cell lines, HSPA8, NLC, NPM1, PLEC, and VIM expression were upregulated by each of the three APDs (Additional file 2: Figure S3, Additional file 3: Figure S4, Additional file 4: Figure S5, Additional file 5: Figure S6, Additional file 6: Figure S7, Additional file 7: Figure S8, Additional file 8: Figure S9, Additional file 9: Figure S10, Additional file 10: Figure S11, Additional file 11: Figure S12, Table 3). In contrast to the increase in HIST1H4B expression induced by haloperidol and risperidone, clozapine reduced HIST1H4B expression in both C6 and B35 cells (Additional file 12: Figure S1 and Additional file 13: Figure S2). Moreover, HIST1H4B, NLC, and NPM1 were expressed in cell nuclei. HSPA8 was generally detected throughout cells. The expression of NLM and NPM1 was induced and specifically accumulated in cell nuclei. PLEC and VIM were mainly expressed in cell nuclei, but were also detected in the cell cytoplasm. Furthermore, we also observed accumulation of PLEC and VIM staining on the nuclear membrane of APD-treated cells.

**Table 3:** Immunofluorescent staining revealed alterations in protein expression in APD-treated C6 and B35 cells

	C6			B35		
	HAL	RIS	CLO	HAL	RIS	CLO
HIST1H4B	**↑**	**↑**	**↓**	**↑**	**↑**	**↓**
HSPA8	**↑**	**↑**	**↑**	**↑**	**↑**	**↑**
NLC	**↑**	**↑**	**↑**	**↑**	**↑**	**↑**
NPM1	**↑**	**↑**	**↑**	**↑**	**↑**	**↑**
PLEC	**↑**	**↑**	**↑**	**↑**	**↑**	**↑**
VIM	**↑**	**↑**	**↑**	**↑**	**↑**	**↑**

**“↑”** means induction of expression compared to control group; “**↓**” means reduction of expression compared to control group

### Regulation of protein expression in sub-chronic and chronic APD-treated rat cortices

**Fig. 2:**
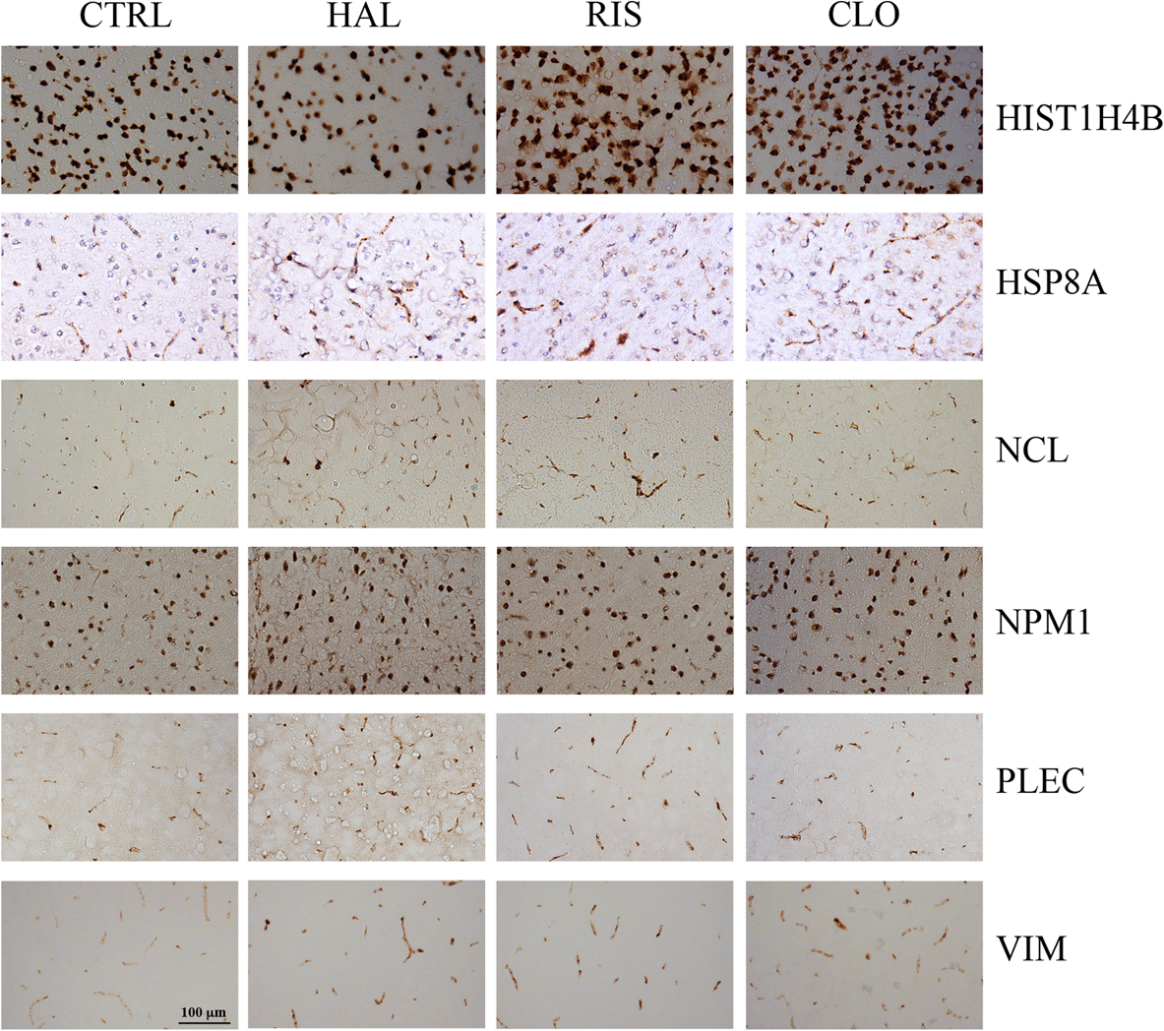
Immunohistochemical staining of the rat prefrontal cortex following sub-chronic (1 week) treatment with APDs

**Fig. 3:**
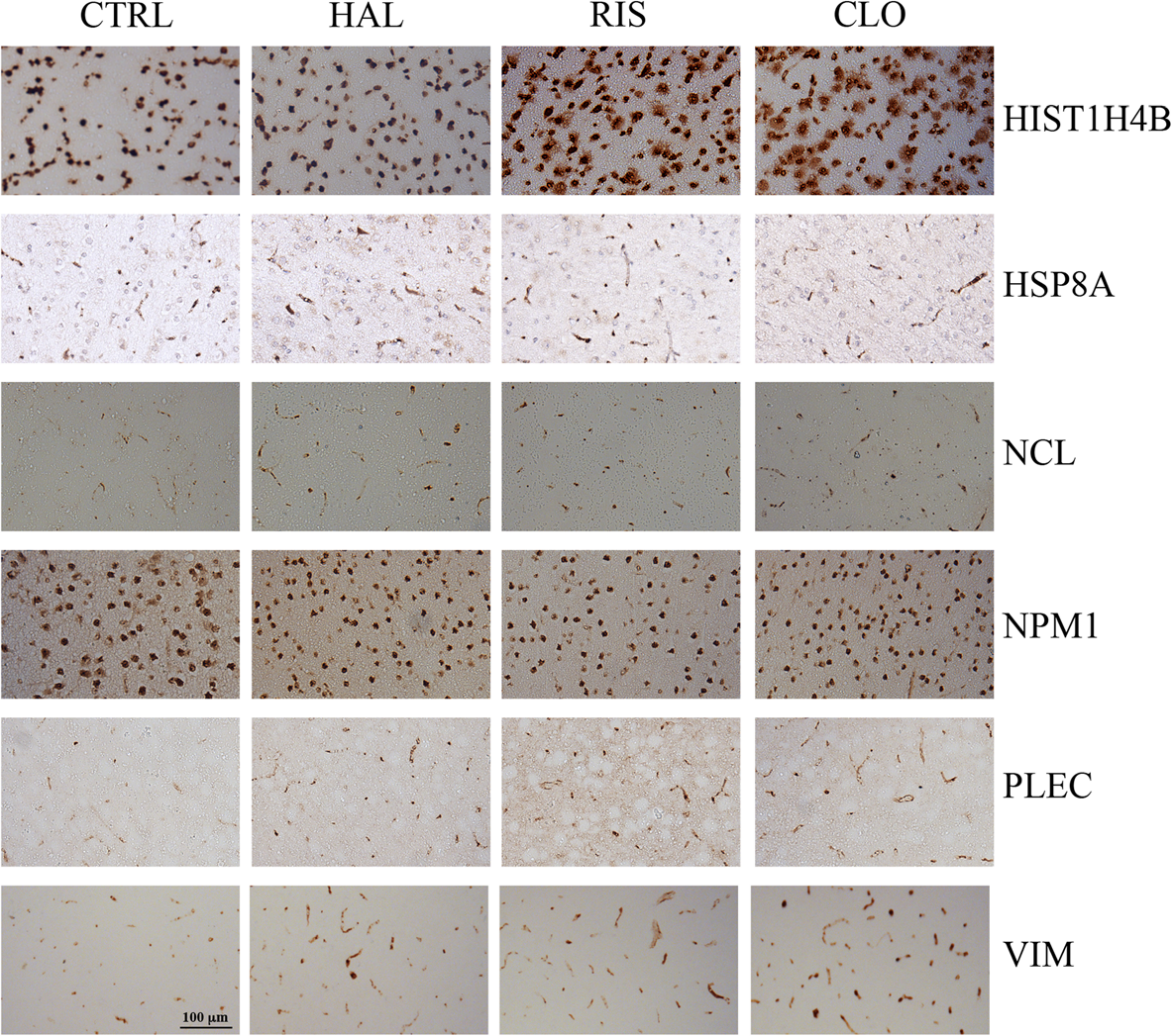
Immunohistochemical staining of the rat prefrontal cortex following chronic (4-weeks) treatment with APDs

We also compared the regulation of protein expression in sub-chronic and chronic APD-treated rat cortices using immunohistochemistry. As shown in Fig. 2, each of the three APDs induced HSP8A, NLC, NPM1, PLEC, and VIM expression in the cortex of sub-chronic APD-treated rats (Additional file 14: Table S2). Haloperidol and risperidone did not significantly change HIST1H4B expression in the rat cortex. Clozapine reduced HIST1H4B expression in the rat cortex. In the chronic APD-treated rat cortex, NLC, PLEC, and VIM expression were induced by each of the three APDs (Fig. 3, Additional file 14: Table S2). Haloperidol and risperidone did not affect HIST1H4B expression, and clozapine reduced HIST1H4B expression in the chronic APD-treated rat cortex. Moreover, none of the three APDs altered HSPA8 and NPM1 expression in the chronic APD-treated rat cortex.

### Real-time quantitative PCR revealed alterations in gene expression in sub-chronic APD-treated C6 and B35 cells

Levels of the HIST1H4B, HSPA8, NCL, NPM1 PLEC and VIM transcripts were measured to investigate the effect of APDs on the expressions of genes encoding the proteins discovered in this study. As shown in Table 4, clozapine, but not haloperidol or risperidone, increased Hist1h4b expression in C6 cells. All three APDs decreased Hspa8, Npm1 and Plec expression in C6 cells. Ncl and Vim expression were not significantly affected by the three APDs in C6 cells. In B35 cells, risperidone and clozapine increased Hist1h4b expression. None of the three APDs affected Hspa8 and Ncl expression in B35 cells. Npm1 expression was increased by haloperidol and decreased by clozapine treatment in B35 cells. Plec and Vim expression were increased by clozapine in B35 cells.

**Table 4:** Relative expression of Hist1h4b, Hspa8, Ncl, Npm1, Plec, and Vim genes in C6 and B35 cells after sub-chronic treatment by haloperidol, risperidone, or clozapine

		C6						B35					
Gene	Group	Fold Mean ± SD	*p*-valne^**a**^	MD	*F*	df	ANOVA-*p*	Fold Mean ± SD	*p*-valne	MD	*F*	df	ANOVA-*p*
Hist1h4b	CTRL	1.00 ± 0.183			8.374	12	0.003	1.00 ± 0.199			22.561	12	0.000
	HAL	1.67 ± 0.091	0.764	0.6748				2.23 ± 0.629	0.198	1.2308			
	RIS	0.90 ± 0.811	0.999	−0.0961				3.03 ± 0.778	0.024 ^**b**^	2.0262*			
	CLO	4.56 ± 2.221	0.003^**b**^	3.5581*				6.17 ± 1.554	0.000 ^**b**^	5.1730*			
Hspa8	CTRL	1.00 ± 0.063			298.9	12	0.000	1.00 ± 0.196			0.690	12	0.575
	HAL	0.13 ± 0.042	0.000 ^**b**^	−0.8703*				0.84 ± 0.196	0.808	− 0.1624			
	RIS	0.08 ± 0.039	0.000 ^**b**^	−0.9187*				1.10 ± 0.514	0.938	0.1023			
	CLO	0.20 ± 0.053	0.000 ^**b**^	−0.8039*				0.84 ± 0.232	0.804	−0.1638			
Ncl	CTRL	1.00 ± 0.183			1.362	12	0.301	1.00 ± 0.420			1.347	12	0.306
	HAL	0.35 ± 0.185	0.162	−0.6501				1.64 ± 0.780	0.197	0.6423			
	RIS	0.74 ± 0.745	0.763	−0.2626				1.36 ± 0.333	0.598	0.3634			
	CLO	0.63 ± 0.481	0.556	−0.3673				1.14 ± 0.204	0.957	0.1374			
Npm1	CTRL	1.00 ± 0.118			4.778	12	0.020	1.00 ± 0.077			17.643	12	0.000
	HAL	0.39 ± 0.235	0.000 ^**b**^	−0.6146*				1.65 ± 0.374	0.005 ^**b**^	0.6497*			
	RIS	0.49 ± 0.381	0.000 ^**b**^	−0.5104*				0.79 ± 0.059	0.467	−0.2109			
	CLO	0.52 ± 0.192	0.000 ^**b**^	−0.4820*				0.49 ± 0.264	0.024 ^**b**^	−0.5105*			
Plec	CTRL	1.00 ± 0.140			44.67	12	0.000	1.00 ± 0.120			27.378	12	0.000
	HAL	0.05 ± 0.040	0.000 ^**b**^	−0.9482*				0.59 ± 0.079	0.052	−0.4090			
	RIS	0.13 ± 0.147	0.000 ^**b**^	−0.8690*				0.99 ± 0.220	1.000	−0.0064			
	CLO	0.14 ± 0.170	0.000 ^**b**^	−0.8556*				1.93 ± 0.347	0.000 ^**b**^	0.9341*			
Vim	CTRL	1.00 ± 0.183			2.002	12	0.167	1.00 ± 0.077			11.822	12	0.001
	HAL	1.36 ± 0.549	0.453	0.36478				1.72 ± 0.365	0.796	0.7236			
	RIS	0.78 ± 0.412	0.769	−0.22386				1.55 ± 0.265	0.891	0.5534			
	CLO	1.33 ± 0.359	0.521	0.3327				6.07 ± 2.688	0.001 ^**b**^	5.0720*			

Data are presented as mean of expression fold change ± SD. All gene expression experiments were performed in biological duplicates. SD = standard deviation;

MD = mean difference; df = degree of freedom; ANOVA-*p* = *p*-value from one-way ANOVA analysis. ^**a**^*p*-value = significant value calculated from Dunnett’s C-tests that compare each APD-treated group to control group. ^**b**^ *p*-value < 0.05. *The mean difference is significant at the 0.05 level in Dunnett’s C-tests

## Discussion

APDs induce nuclear protein regulation in B35 neuronal cells, C6 glial cells and also in the rat cortex. We only identified 25 proteins in this study, even though we used iTRAQ reagent coupled with LC/MS/MS to discover effects on proteins in the nuclei of APD-treated B35 cells. The number of the proteins identified in this study is quite low compared to other LC/MS/MS-based proteomic analyses. In this study, we used a 3200 Q TRAP LC/MS/MS instrument as the detection system to identify iTRAQ labelled peptides. The sensitivity of the equipment we used extremely limited the detection of protein samples. This limited sensitivity is the major reason why we detected a low number of proteins in this proteomic experiment. Also, the use of nuclear proteins as the restricted sample source to reduce the sample complexity is another reason why we identified few proteins in this study.

Histones are found in eukaryotic cells and package DNA into nucleosomes. Histones also play important roles in DNA condensation and gene regulation. Histone modifications have been suggested to be related to the pathogenesis of schizophrenia. Some previous studies have reported increased histone deacetylase (HDAC1) expression in postmortem brain samples from patients with schizophrenia [19, 20]. Decreased expression of the reelin gene is associated with increased methylation of CpG islands in the reelin promoter [21], and reduced acetylation of histones [22] in NT2 cultures. HDAC inhibition has been proposed to improve neurodegeneration and cognitive function, and to induce neurogenesis in animals [23, 24]. HDAC2 overexpression has been shown to reduce the dendritic spine density, synapse number, and synaptic plasticity of neurons [25]. Some conflicting studies have shown that siRNA- and drug-induced HDAC1 inhibition impairs learning and increases cell death in rats. Moreover, increased HDAC1 expression protects rats from ischemic cell death and DNA damage [26]. In addition, as shown in the study by Akbarian and his colleagues, haloperidol and risperidone induce histone H3 phospho-acetylation, which is reversed by MK-801 [27]. A similar finding was also reported by Alessandra, who showed that haloperidol increased phosphorylation of histone H3 on serine28 (H3S28) [28]. In the present study, haloperidol and risperidone increased HIST1H1C, H3F3B and HIST1H4B expression. Clozapine reduced H3F3B and HIST1H4B expression, but not HIST1H1C expression. Although the results in this study might simply infer that APDs regulate histone expression to further modulate chromatin formation and gene regulation, histone modifications are simply the critical biological processes for mediating chromatin formation. Further examinations should be performed to clarify the relations between APD-induced regulation of histone expression and histone modifications.

HSPA8 and NPM1 are chaperone proteins that were affected by each of the three APDs in both B35 and C6 cells. APD-induced expression of HSPA8 and NPM1 was also observed in the sub-chronic APD-treated rat cortex. According to a recent study, a polymorphism in the HSPA8 gene (rs1136141) in patients with first-episode psychosis served as a risk factor for psychosis development [29]. Proteomic and epigenetic studies of postmortem brains also revealed downregulation of HSPA8 in patients with schizophrenia [10, 30], as well as in patients with Alzheimer’s disease (AD) [31]. Another study also revealed downregulation of HSPA8 and NPM1 within layer 2 of the insular cortex, which is closely related to auditory hallucinations and language, in patients with schizophrenia [32]. A microarray study revealed downregulation of NPM1 gene expression in the postmortem cortex of patients with schizophrenia [33]. Based on our data, APD-induced HSP8A and NPM1 expression might be implicated in the possible therapeutic effects of these drugs on treating schizophrenia. As shown in a recent proteomic study, HSPA8 and NPM1 expression are reduced in oligodendrocytes following an acute clozapine treatment and subsequently restored by MK-801 [34]. In our previous research, acute (less than 3 days) APD-induced downregulation of protein expression might be oppositely regulated by a sub-chronic (more than 3 days) APD treatment [35]. This difference might be the reason for the inconsistent APD-induced alternations in the expression of the HSPA8 and NPM1 proteins between the two studies. Interestingly, none of the three APDs modulated HSPA8 and NPM1 expression in the chronic APD-treated rat cortex. This finding is explained by the observation that normal expression levels of HSPA8 or NPM1 should be maintained for these proteins to function as important chaperones in living animals, arguing against chronic APD-induced changes in protein expression. We also propose that living animals may try to maintain the original HSPA8 and NPM1 chaperone functions through various regulatory processes in the brain that prevent changes in protein expression induced by environmental stress.

NPM1 and NCL are multifunctional proteins in cells. NPM and NCL transit to the nucleoplasm in response to stress and participate in the repair of DNA damages [36]. NPM1 and NCL also regulate the expression of proteins related to the MAPK signalling pathway [37], which is abnormally expressed in the anterior cingulate (ACC) and dorsolateral prefrontal cortex (DLPFC) of patients with schizophrenia [38, 39]. We postulate that increased expression of NPM1 and NCL might be implicated in the regulation of proteins related to the MAPK signalling pathway when patients with schizophrenia are treated with certain APDs.

The VIM mRNA levels in superficial, deep, and white matter layers of the anterior cingulate gyrus were not significantly changed in patients with schizophrenia in a previous study [40]. Moreover, VIM expression is not affected by haloperidol or clozapine [41] treatment in rats. In addition, the VIM expression level in postmortem brain samples did not differ between patients with schizophrenia and control groups. VIM interacts with actin filaments through its C-terminal residue and interacts with the actin cytoskeleton through PLEC [42, 43]. In several cell types, PLEC also plays an important role in linking actin microfilaments, microtubules and intermediate filaments [42] to maintain cell morphogenesis and cell plasticity. Schizophrenia has been proposed as a disease with abnormal neuronal plasticity and neuronal regeneration in the brain. APDs were thought to induce plasticity in neuronal cells. All three APDs used in this study increased PLEC and VIM expression in both B35 and C6 cells. We also observed increased expression of PLEC and VIM in the cortex of sub-chronic and chronic APD-treated rats. Several publications have reported a lack of change in VIM expression in patients with schizophrenia. Anchoring of PLEC and VIM on the nuclear membrane is also a critical step in the reorganization ofactin microfilaments, microtubules and intermediate filaments. We also observed an accumulation of PLEC and VIM staining on the nuclear membrane of APD-treated B35 and C6 cells in this study. We propose that APDs-induced modulation of cell plasticity is associated with increased expression of PLEC and VIM. Furthermore, glutamate receptor subunit 3A(NR3A) has been suggested to interact with PLEC to function in intracellular processes, including trafficking and targeting of NR3A-containing receptors and PKC activation [44, 45]. Abnormal NR3A expression has also been observed in patients with various psychiatric disorders, including schizophrenia [46, 47]. Thus, APD-induced PLEC modulation may play important roles in the pathogenesis of schizophrenia by affecting NR3A expression and glutamate receptor function.

In this study, we also examined the regulations of the expression of the Hist1h4b, Hspa8, Ncl Npm1, Plec and Vim genes using real-time quantitative PCR. Compensatory regulation of Hist1h4b expression was observed in clozapine-treated C6 and B35cells. We also revealed compensatory regulation of Hspa8, Npm1 and Plec expression in APD-treated C6 cells. Furthermore, compensatory regulation of Npm1 expression was also investigated in B35 cells treated with clozapine. The compensatory regulation of these genes suggested that the expression of these proteins might not be transcriptionally regulated at the mRNA level in APD-treated cells. Although some proteins whose expression was induced by APDs in this study showed similar trends in gene expression at the transcriptional level, additional examinations should be performed to further verify the relations between APD-induced alterations in protein expression and the regulation of gene expression.

To clarify whether the alterations in protein expression found in this study are APDs specific or not, we examined NCL expression in C6 and B35 cells treated sub-chronically with all-trans retinoic acid (ATRA, 20 μM), H_2_O_2_ (40 μM), or caffeic acid (CA, 10 μM). As shown in Additional file 15: Figure S13(a), ATRA slightly induced NCL expression while H_2_O_2_ and CA dramatically induced NCL expression in C6 cells from two independent treatment batches. Moreover, ATRA reduced NCL expression but H_2_O_2_ and CA slightly induced NCL expression in B35 cells from two independent treatment batches. We also observed induction of VIM expression in ATRA treated C6 (Additional file 15: Figure S13(b)). H_2_O_2_ and CA did not significantly regulate VIM expression in C6 cells. ATRA, H_2_O_2_, and CA induce VIM expression in B35 cells. These results suggest that expression changes of NCL induced by APDs in both cells are not APDs specific effects. Also, most of the proteins identified in this study are the downstream proteins in different signaling pathways and to be regulated by various proteins at the same time in cells. We propose that the regulatory effects on these proteins might be similar between APDs but might not be APDs specific.

Based on our LC/MS/MS data, SLC25A5, HIST1H1C, H3F3B, NONO, LMNA and RPS3A expression were regulated in APD-treated B35 cells. HIST1H4B was chosen as a representative of histone protein expression in the follow-up validation experiments. The SLC25A5, NONO, LMNA and RPS3A proteins were not chosen for validation because little information about the relation between schizophrenia and these proteins is available. Although this study is the first to show that APDs (haloperidol, risperidone, and clozapine) regulate the expression of SLC25A5, NONO, LMNA and RPS3A in B35 cells, further studies should be performed to clarify the roles of these proteins in the pathogenesis of schizophrenia and the mechanisms of action of these antipsychotic drugs. As shown in the immunofluorescent staining of B35 and C6 cells, HSPA8 and VIM were expressed in both the cell nucleus and cytoplasm. This distribution might be the reason for the inconsistent results obtained using immunofluorescent staining and western blots in APD-treated C6 cells.

## Conclusions

We identified 12 proteins expressed in nuclei of B35 cells that were regulated by the APDs used in this study. We used several experimental methods to validate APD-induced alternation in HIST1H4B, HSPA8, NCL, NPM1, PLEC, and VIM expression in different cell types (B35 and C6 cells) and also in the rat cortex. APD-induced alterations in HSPA8 and VIM expression differed between C6 cells and B35 cells. APD-induced alterations in HSPA8 and PLEC expression varied over time in the rat cortex.

## Additional files

Additional file 1: **Table S1.** Reference sequences and primer sequences used to analyse gene expression in real-time quantitative PCR experiments. (TIFF 983 kb)

Additional file 2: **Figure S3.** Immunofluorescent staining revealed alterations in HSPA8 expression in APD-treated C6 cells. (TIFF 899 kb)

Additional file 3: **Figure S4.** Immunofluorescent staining revealed alterations in HSPA8 expression in APD-treated B35 cells. (TIFF 501 kb)

Additional file 4: **Figure S5.** Immunofluorescent staining revealed alterations in NCL expression in APD-treated C6 cells. (TIFF 666 kb)

Additional file 5: **Figure S6.** Immunofluorescent staining revealed alterations in NCL expression in APD-treated B35 cells. (DOCX 55 kb)

Additional file 6: **Figure S7.** Immunofluorescent staining revealed alterations in NPM1 expression in APD-treated C6 cells. (TIFF 554 kb)

Additional file 7: **Figure S8.** Immunofluorescent staining revealed alterations in NPM1 expression in APD-treated B35 cells. (DOCX 61 kb)

Additional file 8: **Figure S9.** Immunofluorescent staining revealed alterations in PLEC expression in APD-treated C6 cells. (TIFF 641 kb)

Additional file 9: **Figure S10.** Immunofluorescent staining revealed alterations in PLEC expression in APD-treated B35 cells. (TIFF 556 kb)

Additional file 10: **Figure S11.** Immunofluorescent staining revealed alterations in VIM expression in APD-treated C6 cells. (TIFF 732 kb)

Additional file 11: **Figure S12.** Immunofluorescent staining revealed alterations in VIM expression in APD-treated B35 cells. (TIFF 653 kb)

Additional file 12: **Figure S1.** Immunofluorescent staining revealed alterations in HIST1H4B expression in APD-treated C6 cells. (TIFF 570 kb)

Additional file 13: **Figure S2.** Immunofluorescent staining revealed alterations in HIST1H4B expression in APD-treated B35 cells. (TIFF 559 kb)

Additional file 14: **Table S2.** Immunohistochemical staining revealed alterations in protein expression in the rat prefrontal cortex following sub-chronic (1 week) and chronic (4 week) treatment with APDs. (TIFF 377 kb)

Additional file 15: **Figure S13.** Western blot showing ATRA-, H2O2-, and CA- induced changes in the expression of the NCL protein in C6 and B35 cells. Samples were collected from two independent batches of ATRA-, H2O2-, and CA- treated C6 and B35 cells. (TIFF 530 kb)

## Abbreviation

2D-DIGE: 2-dimensional difference gel electrophoresis
2D-PAGE: 2-dimensional polyacrylamide gel electrophoresis
ACC: Anterior cingulate
APDs: Antipsychotic drugs
ATRA: All-trans retinoic acid
CA: Caffeic acid
DLPFC: Dorsolateral prefrontal cortex
EPS: Extrapyramidal symptoms
H3F3B: Histone H3.3
H3S28: Histone H3 on serine28
HDAC1: Histone deacetylase
HIST1H1C: Histone H1.2
HIST1H4B: Histone H4
HSPA8: Heat shock cognate 71 kDa protein
iTRAQ: Isobaric tags for relative and absolute quantitation
LC/MS/MS: Liquid chromatography-tandem mass spectrometry
LMNA: Prelamin-A/C
NCL: Nucleolin
NONO: Non-POU domain-containing octamer-binding protein
NPM1: Nucleophosmin
NR3A: Glutamate receptor subunit 3A
PLEC: Plectin-1
PTMs: Post-translational modifications
RPS3A: 40S ribosomal protein S3a
RT-QPCR: Real-time quantitative polymerase chain reaction
SLC25A5: ADP/ATP translocase 2
VIM: Vimentin
