# Supplementary figures and images for: Proteomic study revealed antipsychotics-induced nuclear protein regulations in B35 cells are similar to the regulations in C6 cells and rat cortex
Source: BMC Pharmacol Toxicol. 2018 Mar 7;19:9. doi: 10.1186/s40360-018-0199-0 (PMC5842604; doi:10.1186/s40360-018-0199-0)

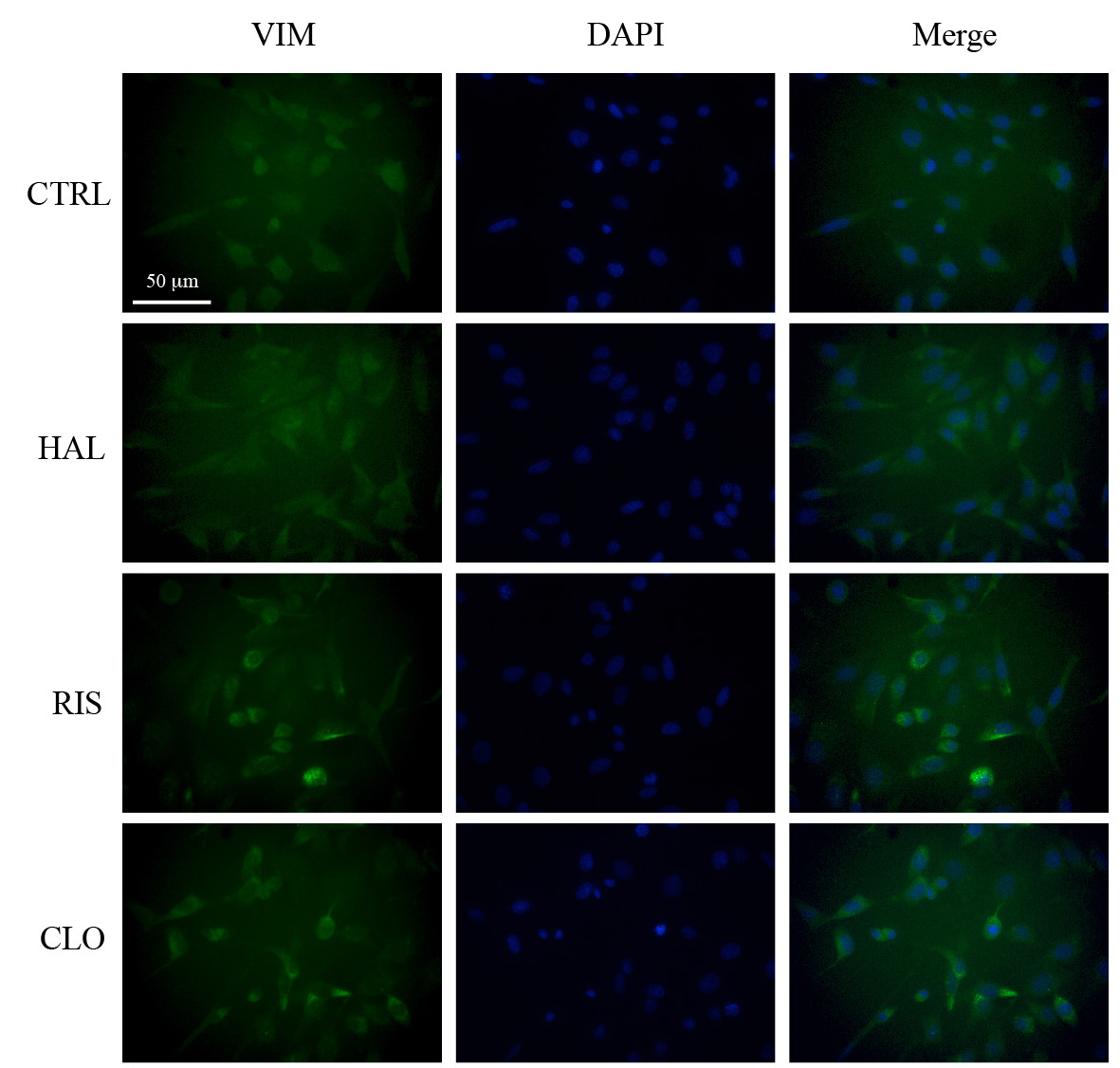

Supplement: Supplementary file 1 — Table S1. Reference sequences and primer sequences used to analyse gene expression in real-time quantitative PCR experiments. (TIFF 983 kb) [file 40360_2018_199_MOESM10_ESM.tif]

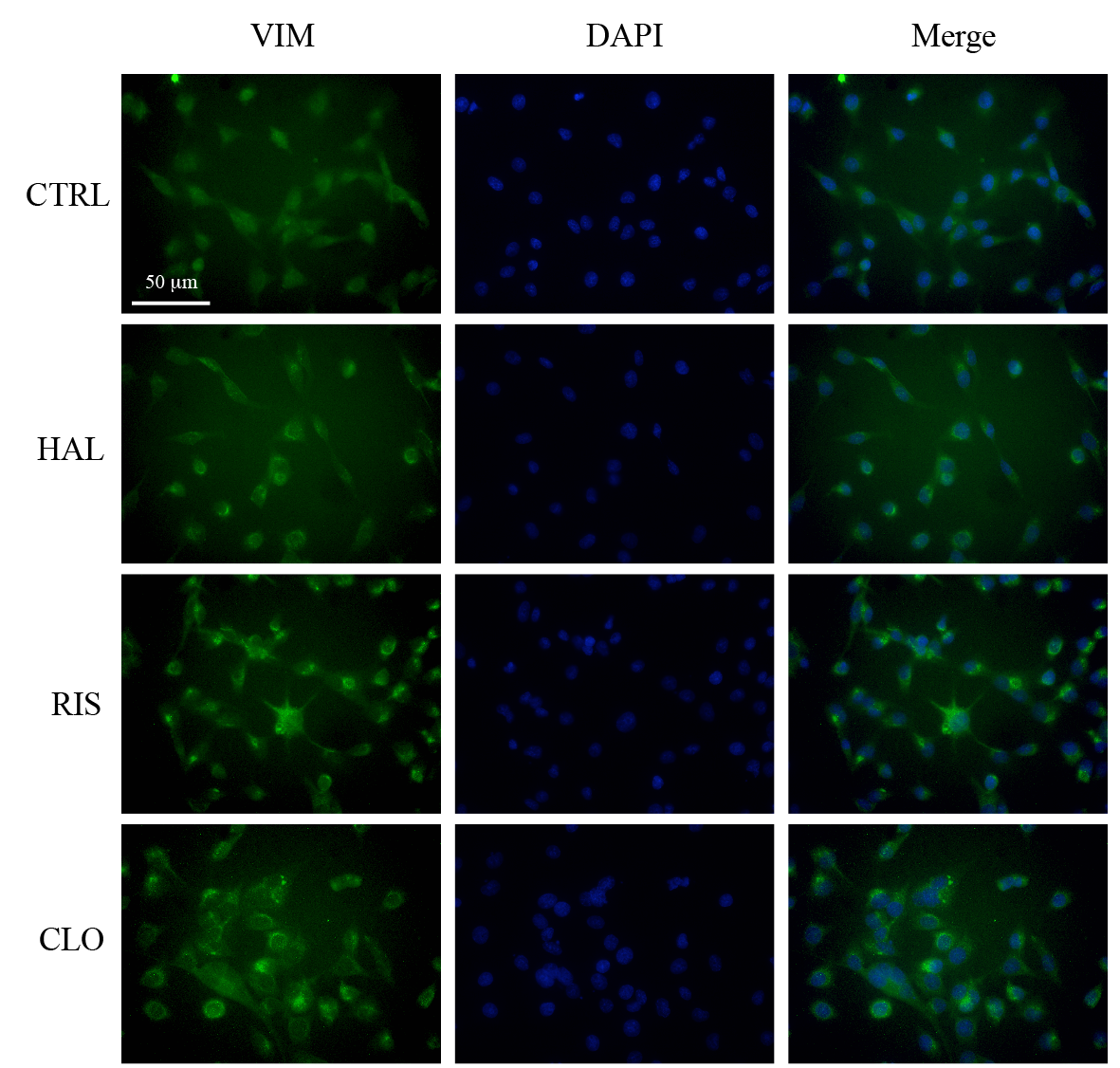

Supplement: Supplementary file 2 — Figure S3. Immunofluorescent staining revealed alterations in HSPA8 expression in APD-treated C6 cells. (TIFF 899 kb) [file 40360_2018_199_MOESM11_ESM.tif]

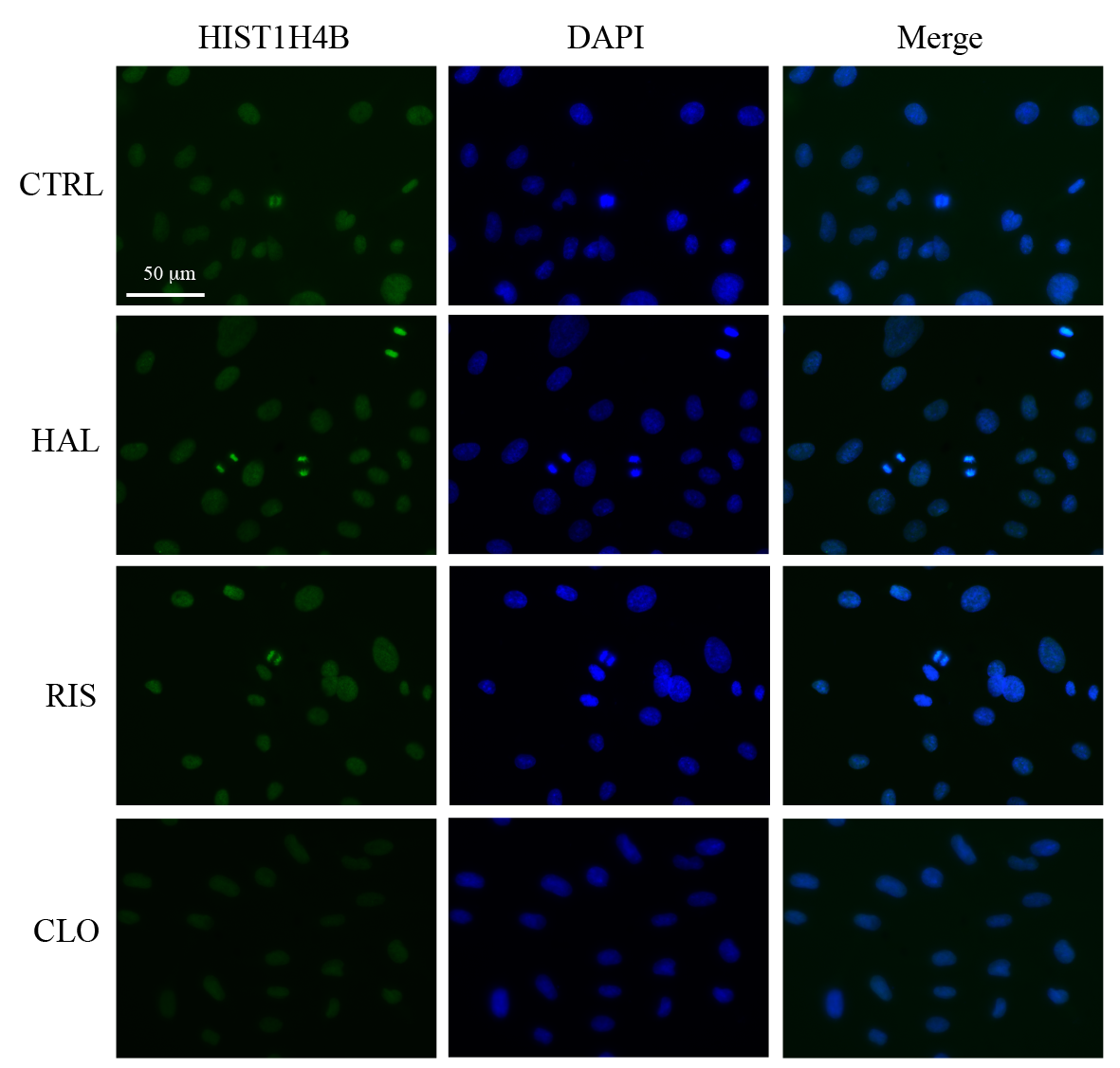

Supplement: Supplementary file 3 — Figure S4. Immunofluorescent staining revealed alterations in HSPA8 expression in APD-treated B35 cells. (TIFF 501 kb) [file 40360_2018_199_MOESM12_ESM.tif]

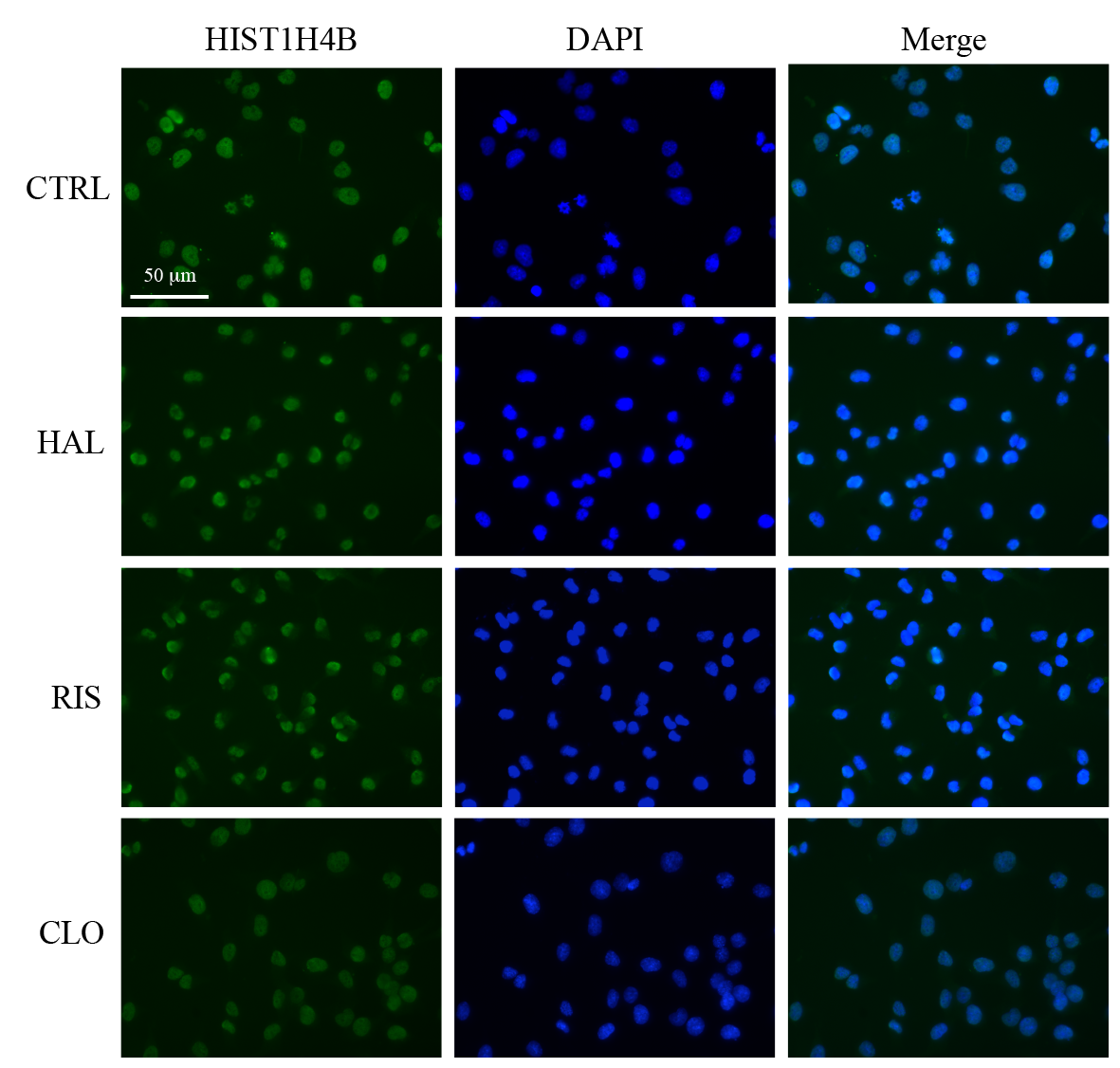

Supplement: Supplementary file 4 — Figure S5. Immunofluorescent staining revealed alterations in NCL expression in APD-treated C6 cells. (TIFF 666 kb) [file 40360_2018_199_MOESM13_ESM.tif]

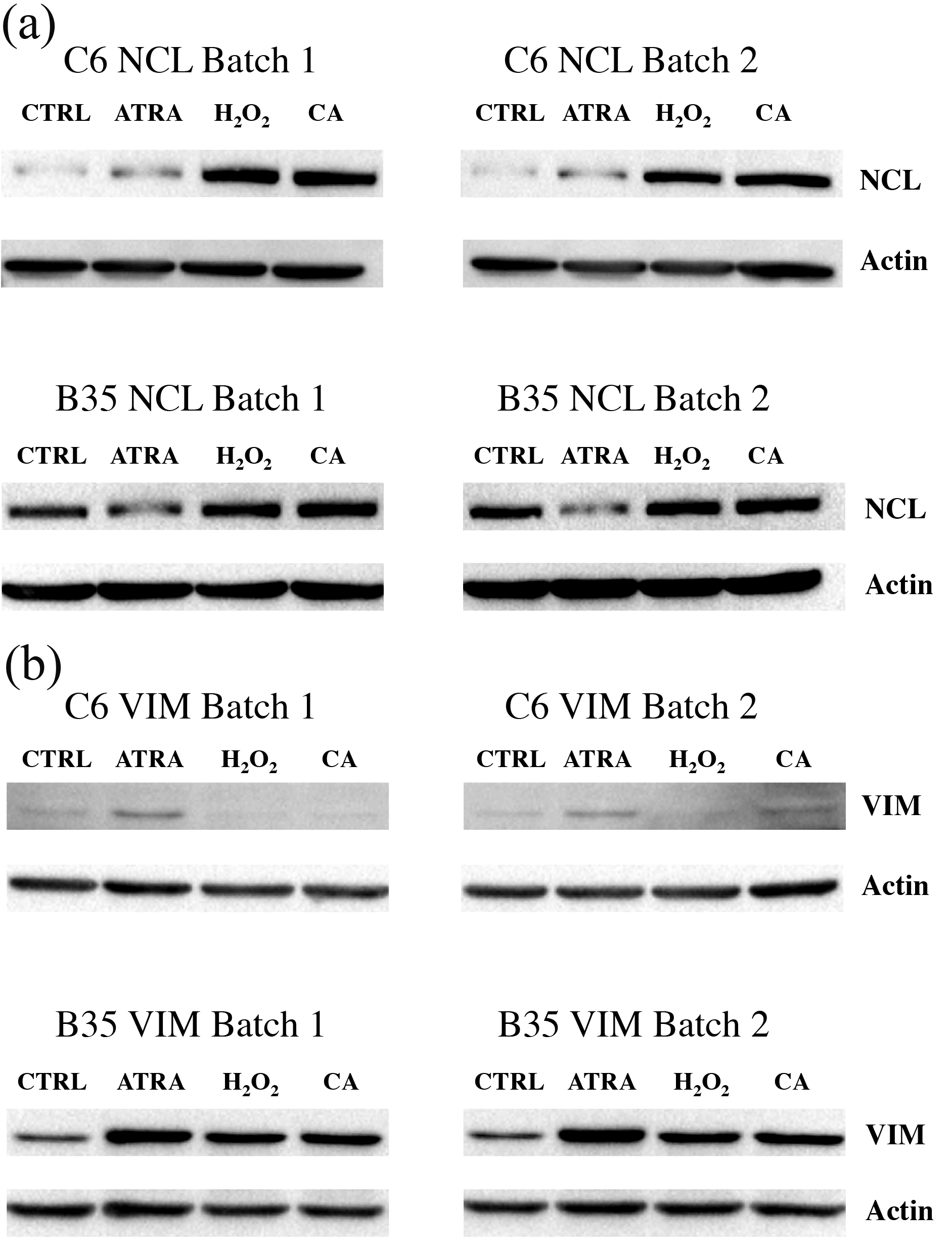

Supplement: Supplementary file 6 — Figure S7. Immunofluorescent staining revealed alterations in NPM1 expression in APD-treated C6 cells. (TIFF 554 kb) [file 40360_2018_199_MOESM15_ESM.tif]

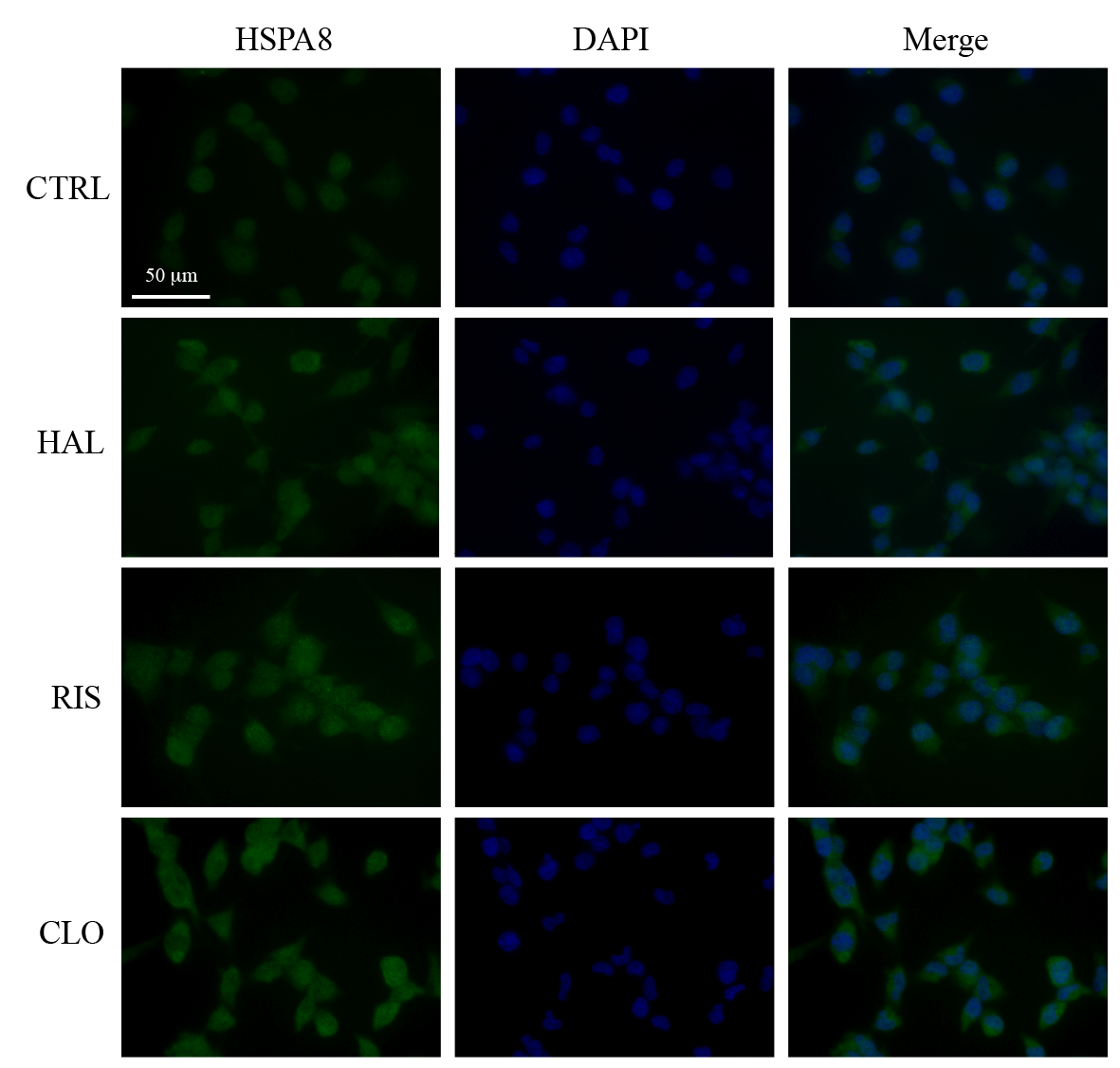

Supplement: Supplementary file 8 — Figure S9. Immunofluorescent staining revealed alterations in PLEC expression in APD-treated C6 cells. (TIFF 641 kb) [file 40360_2018_199_MOESM2_ESM.tif]

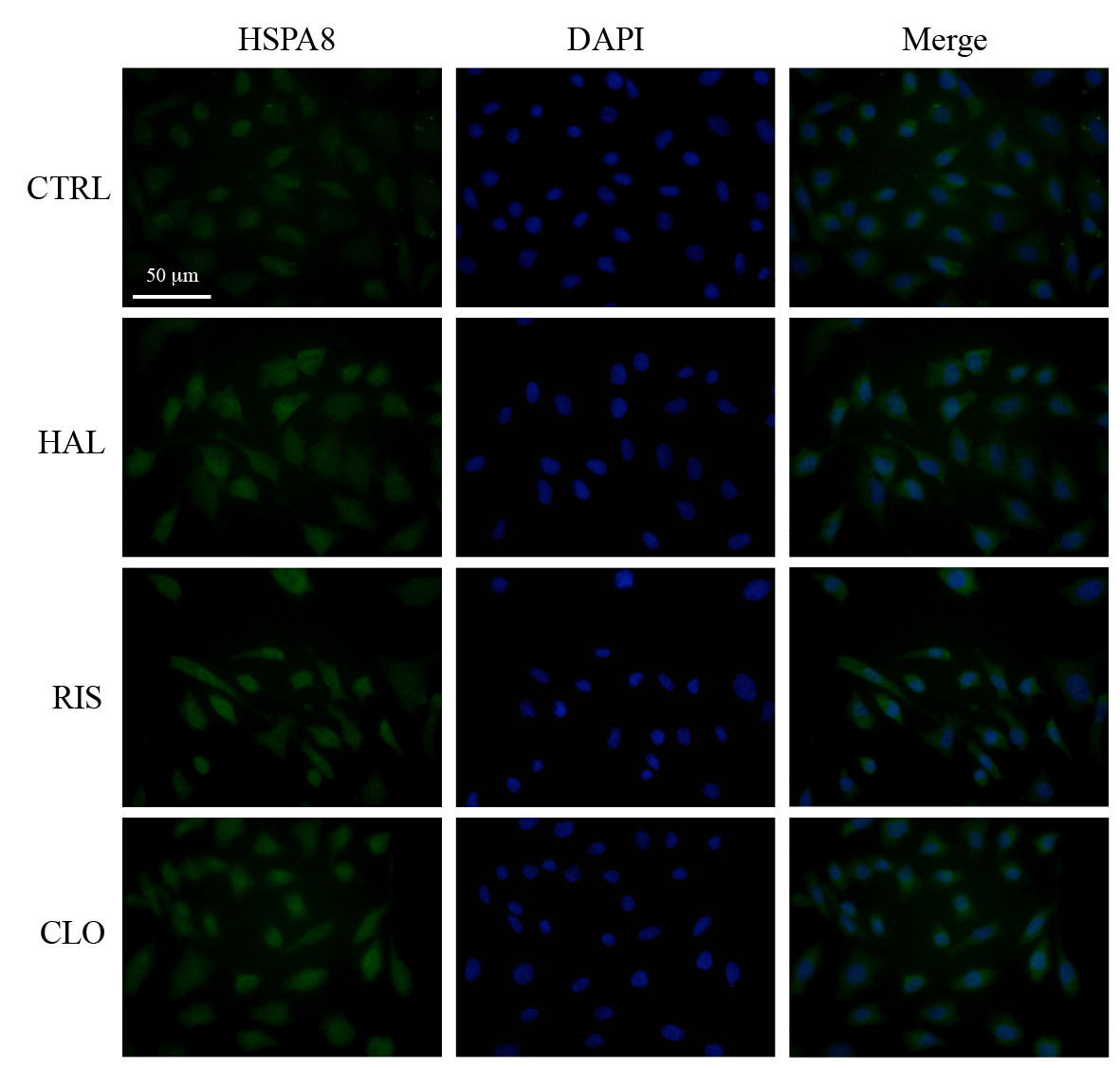

Supplement: Supplementary file 9 — Figure S10. Immunofluorescent staining revealed alterations in PLEC expression in APD-treated B35 cells. (TIFF 556 kb) [file 40360_2018_199_MOESM3_ESM.tif]

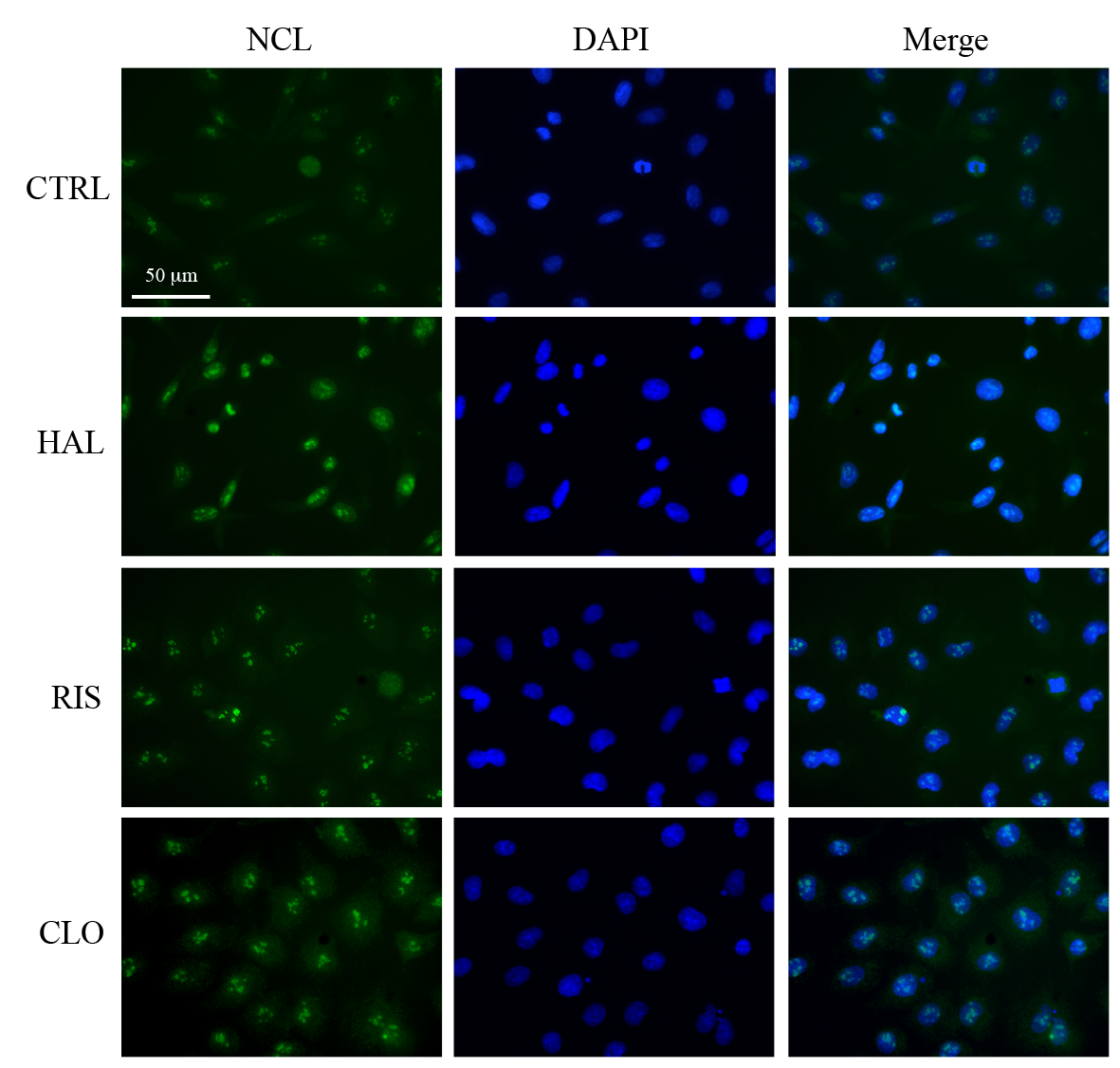

Supplement: Supplementary file 10 — Figure S11. Immunofluorescent staining revealed alterations in VIM expression in APD-treated C6 cells. (TIFF 732 kb) [file 40360_2018_199_MOESM4_ESM.tif]

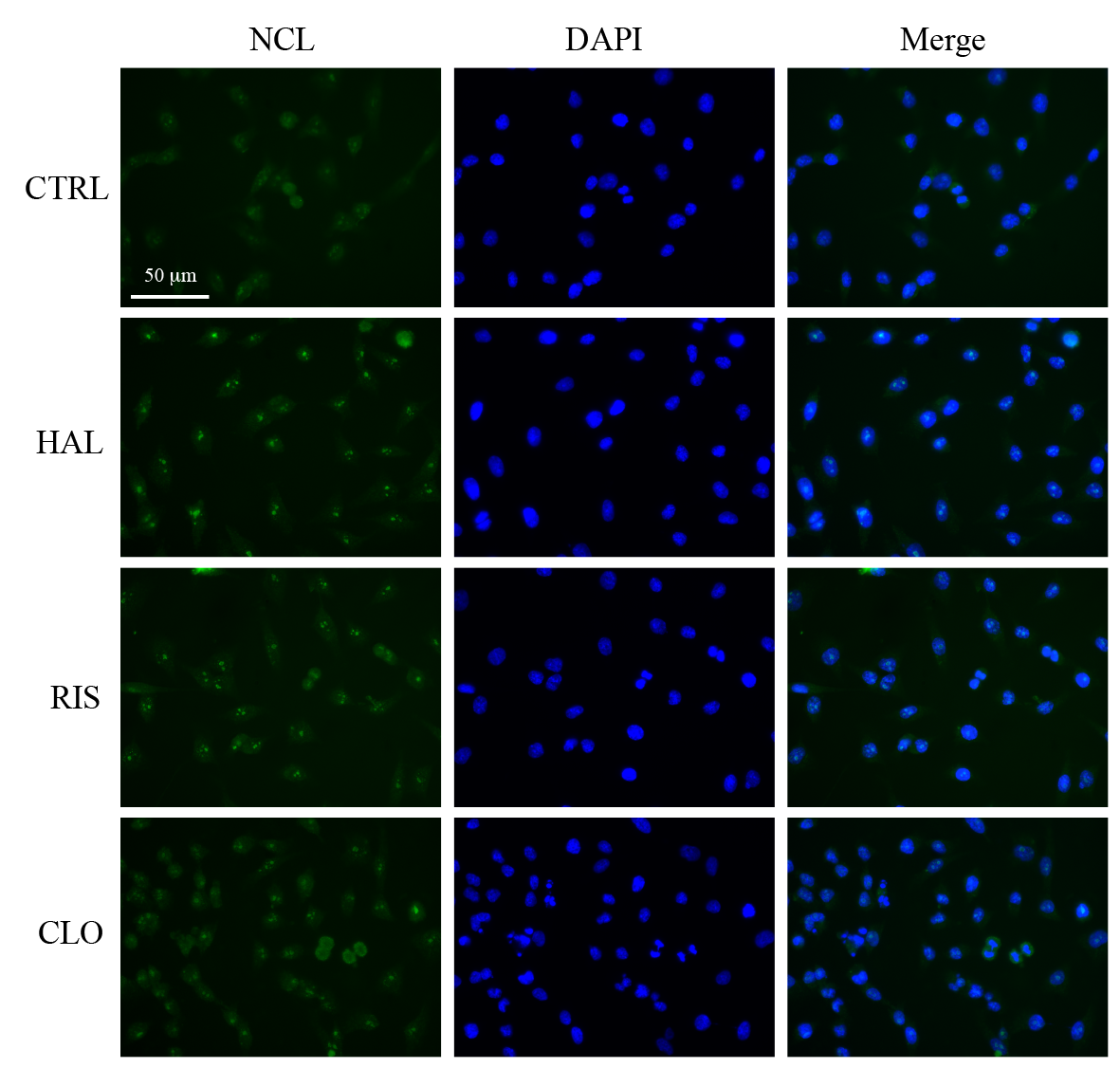

Supplement: Supplementary file 11 — Figure S12. Immunofluorescent staining revealed alterations in VIM expression in APD-treated B35 cells. (TIFF 653 kb) [file 40360_2018_199_MOESM5_ESM.tif]

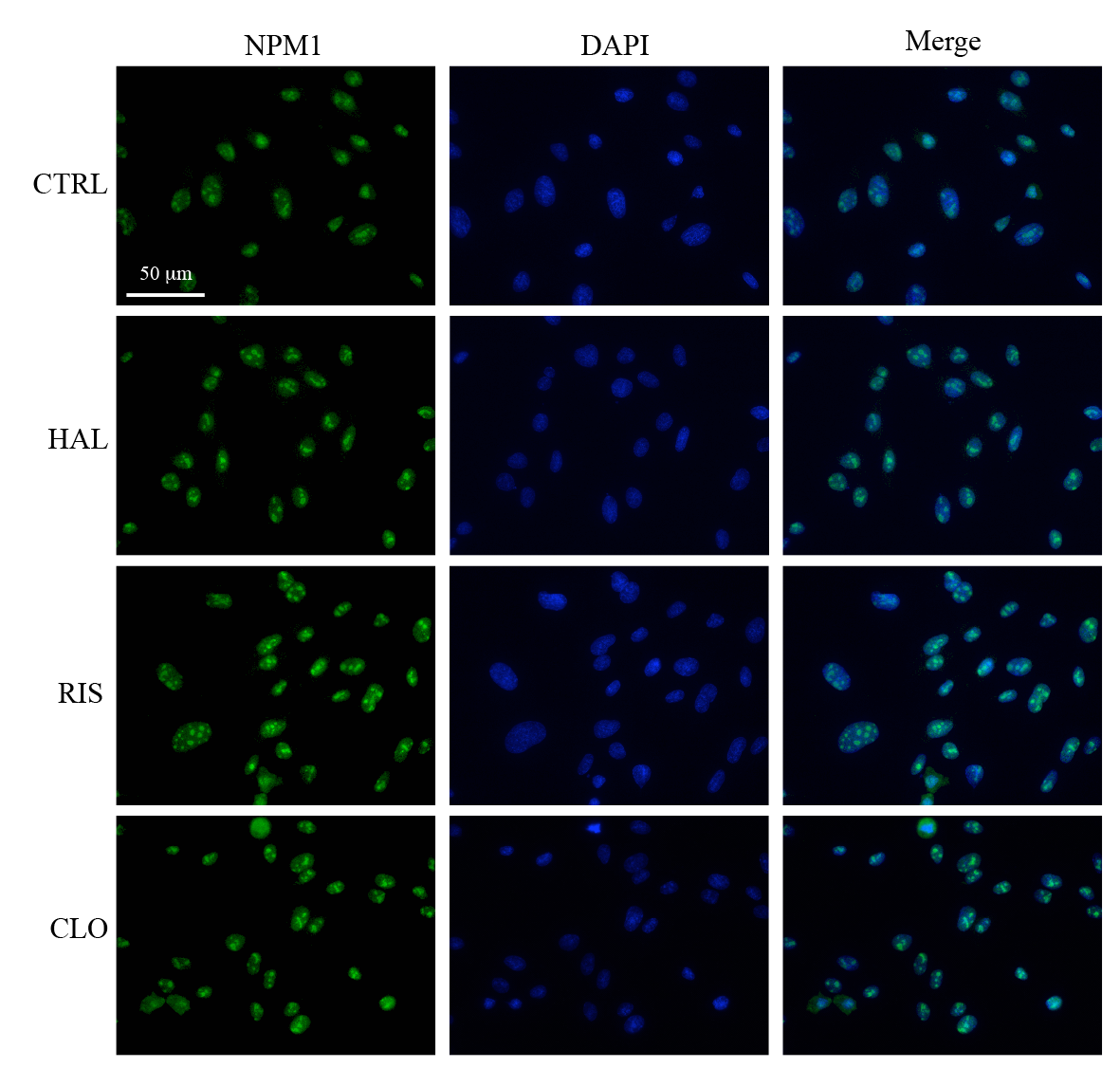

Supplement: Supplementary file 12 — Figure S1. Immunofluorescent staining revealed alterations in HIST1H4B expression in APD-treated C6 cells. (TIFF 570 kb) [file 40360_2018_199_MOESM6_ESM.tif]

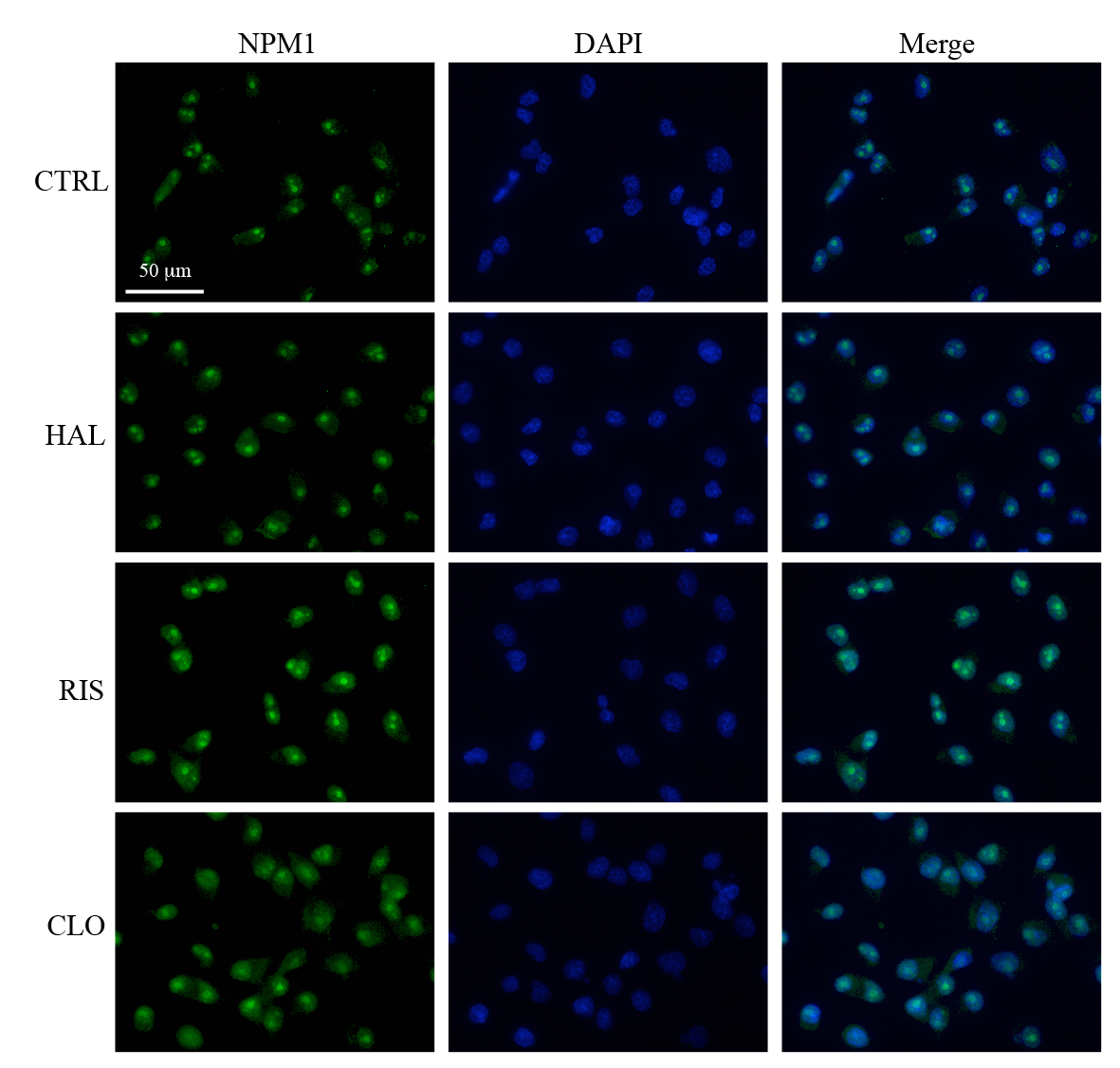

Supplement: Supplementary file 13 — Figure S2. Immunofluorescent staining revealed alterations in HIST1H4B expression in APD-treated B35 cells. (TIFF 559 kb) [file 40360_2018_199_MOESM7_ESM.tif]

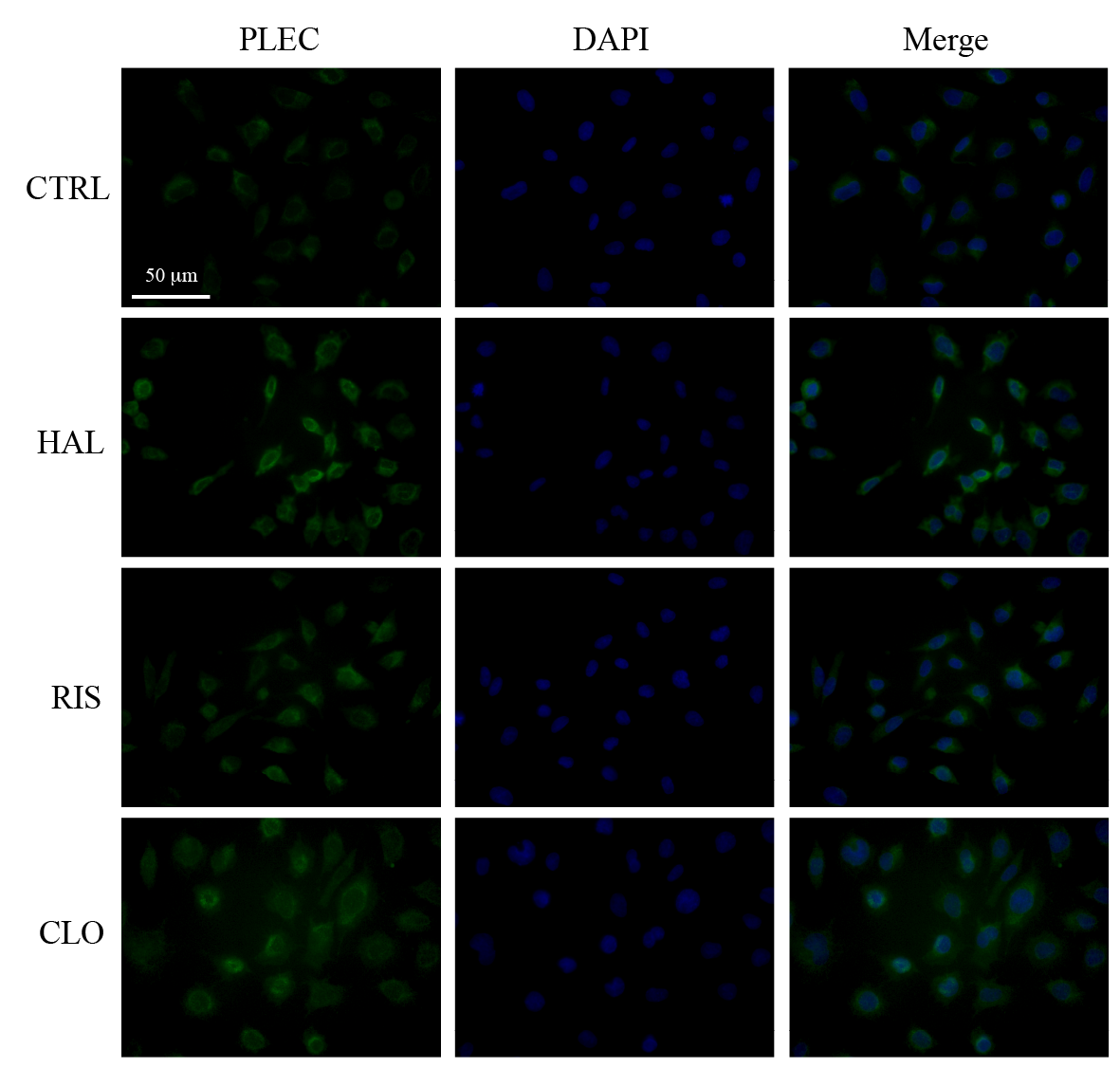

Supplement: Supplementary file 14 — Table S2. Immunohistochemical staining revealed alterations in protein expression in the rat prefrontal cortex following sub-chronic (1 week) and chronic (4 week) treatment with APDs. (TIFF 377 kb) [file 40360_2018_199_MOESM8_ESM.tif]

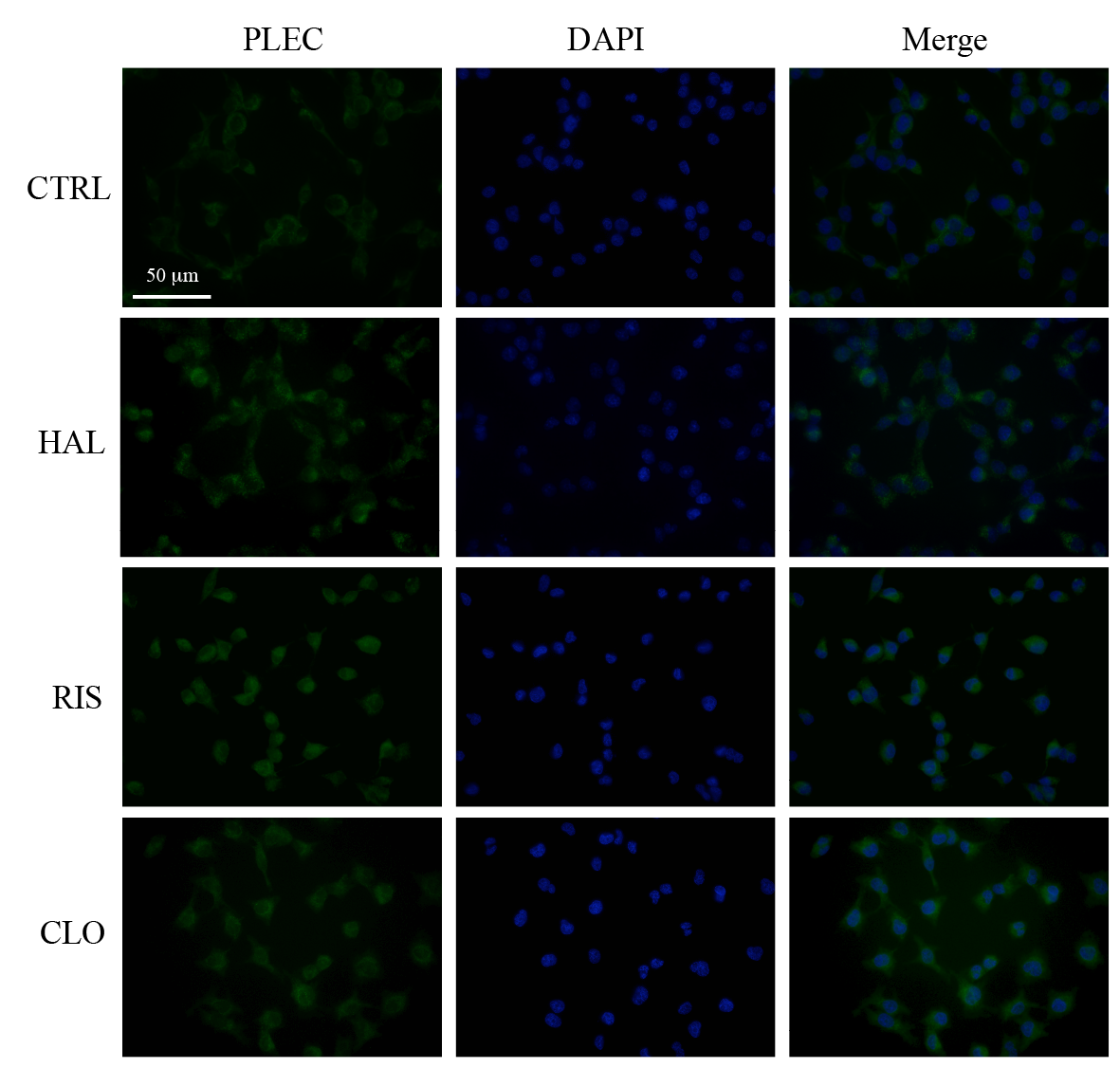

Supplement: Supplementary file 15 — Figure S13. Western blot showing ATRA-, H2O2-, and CA- induced changes in the expression of the NCL protein in C6 and B35 cells. Samples were collected from two independent batches of ATRA-, H2O2-, and CA- treated C6 and B35 cells. (TIFF 530 kb) [file 40360_2018_199_MOESM9_ESM.tif]
