# Supplementary material for: Proteomic study revealed antipsychotics-induced nuclear protein regulations in B35 cells are similar to the regulations in C6 cells and rat cortex
Source: BMC Pharmacol Toxicol. 2018 Mar 7;19:9. doi: 10.1186/s40360-018-0199-0 (PMC5842604; doi:10.1186/s40360-018-0199-0)
Supplement: Supplementary file 5 — Figure S6. Immunofluorescent staining revealed alterations in NCL expression in APD-treated B35 cells. (DOCX 55 kb) [file 40360_2018_199_MOESM14_ESM.docx]

Table S2

Immunohistochemical staining revealed alterations in protein expression in the rat prefrontal cortex following sub-chronic (1 week) and chronic (4 week) treatment with APDs.

|  |  | 1-Week | | |  | 4-Week | | |
| --- | --- | --- | --- | --- | --- | --- | --- | --- |
|  |  | HAL | RIS | CLO |  | HAL | RIS | CLO |
| HIST1H4B |  | NC | NC | **↓** |  | NC | NC | **↓** |
| HSP8A |  | **↑** | **↑** | **↑** |  | NC | NC | NC |
| NLC |  | **↑** | **↑** | **↑** |  | **↑** | **↑** | **↑** |
| NPM1 |  | **↑** | **↑** | **↑** |  | NC | NC | NC |
| PLEC |  | **↑** | **↑** | **↑** |  | **↑** | **↑** | **↑** |
| VIM |  | **↑** | **↑** | **↑** |  | **↑** | **↑** | **↑** |

“**↑**” means induction of expression compared to control group; “**↓**” means reduction of expression compared to control group; NC means no significant change observed.
