# Supplementary material for: Proteomic study revealed antipsychotics-induced nuclear protein regulations in B35 cells are similar to the regulations in C6 cells and rat cortex
Source: BMC Pharmacol Toxicol. 2018 Mar 7;19:9. doi: 10.1186/s40360-018-0199-0 (PMC5842604; doi:10.1186/s40360-018-0199-0)
Supplement: Supplementary file 7 — Figure S8. Immunofluorescent staining revealed alterations in NPM1 expression in APD-treated B35 cells. (DOCX 61 kb) [file 40360_2018_199_MOESM1_ESM.docx]

Table S1

Reference sequences and primer sequences used to analyse gene expression in real-time quantitative PCR experiments.

| Reference Sequence | Primer | Sequence | Product Size (bps) | Annealing Temp.(°C) |
| --- | --- | --- | --- | --- |
| M27443.1 | HIST1H4B-S | GGTGGTAAAGGGCTTGGGAA | 138 | 60 |
|  | HIST1H4B-A | GTAGATGAGGCCGGAGATGC |  |  |
| NM_024351.2 | HSPA8-S | CGCCGAGCTATGTTGCTTTC | 182 | 60 |
|  | HSPA8-A | TGCATCGTTCACCACCATGA |  |  |
| NM_012749.2 | NCL-S | CAAAACCCACGGAGAGTCCA | 159 | 60 |
|  | NCL-A | GCTGGAGTTGTGGTAGCCTT |  |  |
| NM_012992 | NPM1-S | ACCAACAGTTTCCCTTGGGG | 182 | 60 |
|  | NPM1-A | ACTTTGTTACCACCTCCGGG |  |  |
| NM_022401.2 | PLEC-S | CCGCATTGCAGATGAACGAG | 231 | 60 |
|  | PLEC-A | TCGGAGATAGTCCAGGGCAA |  |  |
| NM_031140.1 | VIM-S | GAGGAGATGAGGGAGTTGCG | 205 | 60 |
|  | VIM-A | GGTCAAGACGTGCCAGAGAA |  |  |
